# Costing malaria interventions from pilots to elimination programmes

**DOI:** 10.1186/s12936-020-03405-3

**Published:** 2020-09-14

**Authors:** Katya Galactionova, Mar Velarde, Kafula Silumbe, John Miller, Anthony McDonnell, Ricardo Aguas, Thomas A. Smith, Melissa A. Penny

**Affiliations:** 1 Department of Epidemiology and Public Health, Swiss Tropical and Public Health Institute, Basel, Switzerland; 2 University of Basel, Basel, Switzerland; 3 Malaria Control and Elimination Partnership in Africa at PATH (MACEPA), Lusaka, Zambia; 4 Mahidol Oxford Tropical Medicine Research Unit, Faculty of Tropical Medicine, Mahidol University, Bangkok, Thailand; 5 Centre for Tropical Medicine and Global Health, Nuffield Department of Medicine, University of Oxford, Oxford, UK

**Keywords:** Malaria control, Malaria elimination, Costs, Malaria rapid reporting, IRS, RACD, MDA, Cost models, Resource-allocation, Comparative cost-effectiveness

## Abstract

**Background:**

Malaria programmes in countries with low transmission levels require evidence to optimize deployment of current and new tools to reach elimination with limited resources. Recent pilots of elimination strategies in Ethiopia, Senegal, and Zambia produced evidence of their epidemiological impacts and costs. There is a need to generalize these findings to different epidemiological and health systems contexts.

**Methods:**

Drawing on experience of implementing partners, operational documents and costing studies from these pilots, reference scenarios were defined for rapid reporting (RR), reactive case detection (RACD), mass drug administration (MDA), and in-door residual spraying (IRS). These generalized interventions from their trial implementation to one typical of programmatic delivery. In doing so, resource use due to interventions was isolated from research activities and was related to the pilot setting. Costing models developed around this reference implementation, standardized the scope of resources costed, the valuation of resource use, and the setting in which interventions were evaluated. Sensitivity analyses were used to inform generalizability of the estimates and model assumptions.

**Results:**

Populated with local prices and resource use from the pilots, the models yielded an average annual economic cost per capita of $0.18 for RR, $0.75 for RACD, $4.28 for MDA (two rounds), and $1.79 for IRS (one round, 50% households). Intervention design and resource use at service delivery were key drivers of variation in costs of RR, MDA, and RACD. Scale was the most important parameter for IRS. Overall price level was a minor contributor, except for MDA where drugs accounted for 70% of the cost. The analyses showed that at implementation scales comparable to health facility catchment area, systematic correlations between model inputs characterizing implementation and setting produce large gradients in costs.

**Conclusions:**

Prospective costing models are powerful tools to explore resource and cost implications of policy alternatives. By formalizing translation of operational data into an estimate of intervention cost, these models provide the methodological infrastructure to strengthen capacity gap for economic evaluation in endemic countries. The value of this approach for decision-making is enhanced when primary cost data collection is designed to enable analysis of the efficiency of operational inputs in relation to features of the trial or the setting, thus facilitating transferability.

## Background

The 2018–2030 Global Technical Strategy for malaria declared elimination the ultimate goal for all malaria-endemic countries and outlined a tiered strategy for programmes to transition from control to elimination [1]. An increasing number of countries are moving toward this goal each year [1]. Botswana, Eswatini, Mozambique, Namibia, South Africa, Zambia, and Zimbabwe are leading the way for the African continent [2–6]. For countries where transmission has been reduced to very low levels, progress toward elimination relies on strong surveillance systems to identify and appropriately target areas where transmission is sustained [7]. In addition to universal coverage with vector control and access to preventive and curative interventions, active and reactive responses to clear infection and focal vector control are recommended to find asymptomatic cases and to eliminate the infectious reservoir [7].

Between 2013 and 2015 the Malaria Control and Elimination Partnership in Africa at PATH (MACEPA), together with country programmes, conducted field trials in Zambia [8–11], Senegal [12], and Ethiopia to evaluate the individual and synergistic effects of malaria interventions on transmission interruption. Within these trials, scale-up and optimization of routine malaria preventive interventions were evaluated alongside strategies for malaria surveillance, and both population-wide and focal approaches to clearing parasites from people. MACEPA conducted supervision, monitoring, training, and evaluation activities while the programmes carried out implementation of interventions in communities (i.e. via Community Health Workers (CHWs) or other programme staff). The epidemiological, operational, and economic studies carried out along the MACEPA elimination pilots produced important evidence on the effectiveness of the recommended strategies in these settings [13–16]. Programmes now require tools to transfer and relate findings from these pilots to the specific contexts and the capacity constraints in which they operate in order to formulate adequate strategies toward elimination targets.

Evidence on resource needs and costs informs feasibility of implementation, affordability of the intervention by programmes, appropriate delivery modality, and, when combined with data on effectiveness, allows for comparisons between policy alternatives. However, the relevance of cost estimates obtained from research trials to policy decision-making, is limited by the scalability of piloted interventions, the extent to which economies of scale and scope impact these costs, resource and technical support provided by partners, incentives for trial participation to operational staff and population, and representativeness of the populations targeted [17]. This paper shows how these limitations of field data can be overcome with prospective micro-costing models parameterized with disaggregated data on resource use and prices from trials and secondary sources. Increasing availability of economic data from low and middle-income countries (LMICs) collated in global costing databases and reporting supporting financial requests to partners make this approach attractive for evaluation of new health interventions and deployment strategies [18–20].

To provide further evidence to inform strategic decisions on optimal intervention mixes for malaria elimination, costing models were developed for four key interventions recommended for malaria eliminating countries: malaria rapid reporting (RR), reactive case detection (RACD), mass administration (MDA), and indoor residual spraying (IRS). The paper details application of these models to derive locally and programmatically relevant intervention costs for a reference setting and illustrates how these models could be further extended to cost alternative implementations of interventions in any health system or epidemiological setting, at any desired scale or price level. Sensitivity analyses further inform transferability of the cost estimates derived and guide future cost data collection efforts toward strengthening policy relevance of economic evidence on malaria elimination.

## Methods

The immediate use case for the models presented here is cost-effectiveness or other optimization framework supporting prioritization of interventions within malaria elimination packages. Thus, in developing the methodology, the focus has been on ensuring that *consistent comparisons* could be made between interventions, that the models could be adequately tailored to *different settings*, and that the costs derived reflected *programmatic delivery* of interventions in the African region. Experience of in-country partners in delivering the interventions within the elimination pilots and their subsequent scale-up in the region were first detailed in a reference implementation scenario and then formalized in a costing model defined around that reference scenario.

### Interventions

Four of the MACEPA piloted interventions were evaluated in this study. Malaria RR is defined here as a surveillance intervention that entails weekly reporting of malaria indicators by health facility staff using the District Health Information System 2 (DHIS 2) [21] and a mobile client. RACD refers to reactive focal testing and treatment by CHWs of malaria RDT-positives in the home of an index case (a clinical malaria case identified in either community or health facility) and those in the neighbouring households. MDA describes a strategy for administering anti-malarial drugs by CHWs without prior testing. IRS entails insecticidal spraying of surfaces in inhabited houses; deployment of IRS for elimination is conducted by district teams in a subset of houses targeted by malaria incidence.

### Reference implementation scenarios

The starting point in developing intervention implementation scenarios was global normative guidance. The World Health Organization (WHO) and other partner guidelines (Additional file 1: Table S1) were consulted to define intervention implementation stages and to develop a list of operational activities that take place at each stage (Additional file 1: Table S2). The following activities were costed: (1) “planning”—micro-planning and meetings at central and district levels; (2) “community sensitization”—meetings with community leaders and local authorities and district and community levels, dissemination of information materials, radio and public announcements; (3) “training”—training of supervisors, trainers, and CHWs or facility staff at central and district levels; (4) “procurement, storage, and distribution”—procurement of drugs and supplies including where applicable mobile phones and bikes and expenses related to their storage and distribution to facilities; (5) “programme management and supervision”—programme support at central, regional, district and facility levels, and supervision by appropriate levels of service provision; (6) “implementation”—activities conducted at point of service delivery including reporting; (7) “other”—all other intervention specific activities (i.e. environmental compliance and waste management for IRS). Detailed resource lists were developed for each operational activity specifying resource requirements by programmatic level (i.e. central, regional). These were derived through a process similar to micro-planning, conducted by programmes or trial teams in the planning stages of the project. Unlike programmes, this study drew on guidelines, literature, operational documents from the pilots and inputs from MACEPA staff to acquire an understanding of the context, capacity, service delivery and other programmatic aspects that impact resource requirements for the four interventions.

By comparing operational details across the pilot sites, it was possible to isolate aspects of intervention design that reflected the trial research objectives or were specific to the setting, and features that were common and generalizable to broader geographies outside the pilots. To allow consistent comparisons of costs between interventions, the scope of resources evaluated was standardized across the four interventions and the resource use assumptions were explicitly related to the reference setting. Additionally, assumptions on intensity of activities implemented were aligned both across interventions and over time (i.e*.* greater planning and coordination needs for IRS compared to RR).

### Reference setting

The reference setting was described by a geography summarized with the distance between programmatic levels, scale of implementation that translates to a population target [1 region, 3 districts, 6000 people per health facility catchment area (HFCA)], and programme infrastructure captured with number of health facilities (33 per 10,000 population or an average of 20 HFCAs per district), number of community health workers (1 CHW per 750 people or 8 CHW per HFCA), and access to care [80% (refers to access to any formal health care provider including CHW)]. The *Plasmodium falciparum* parasite all-age prevalence (*Pf*PR) was fixed at 4% and RDT-positivity rate around an index case at 18%. These reference values approximate annual averages from MACEPA trials conducted in Zambia [13].

### Framing of the evaluation

The framing of the costing study formalized in the costing models presented here, reflects the health system’s perspective. The models capture resource use incremental to existing programme infrastructure. Assumptions on capacity to absorb the new intervention were informed by the implementation partner and are reflected in the operational inputs made explicit in the reference scenario. The existing workforce was assumed to be sufficient to deploy the additional interventions at reference levels, capacity constraints then were reflected by how that capacity translated to programmatic outputs (i.e. number of supervision days per campaign round, number of people treated per day by CHW). The models produce both financial and economic costs. The financial costs illustrate the incremental investment needed to deliver the intervention. The economic costs also capture the opportunity cost of using resources, including the health system infrastructure, that were paid through other sources (including wages of programme staff, programme vehicles) or donated (i.e. time of CHWs) [22]. Resource use and costs were traced across the programmatic levels and, comprehensively, throughout the intervention implementation stages. Costs were evaluated over 5 years matching the strategic planning horizon of malaria programmes. All nominal values were expressed in 2014 USD to allow comparisons with the MACEPA trials.

### Costing models

**Fig. 1:**
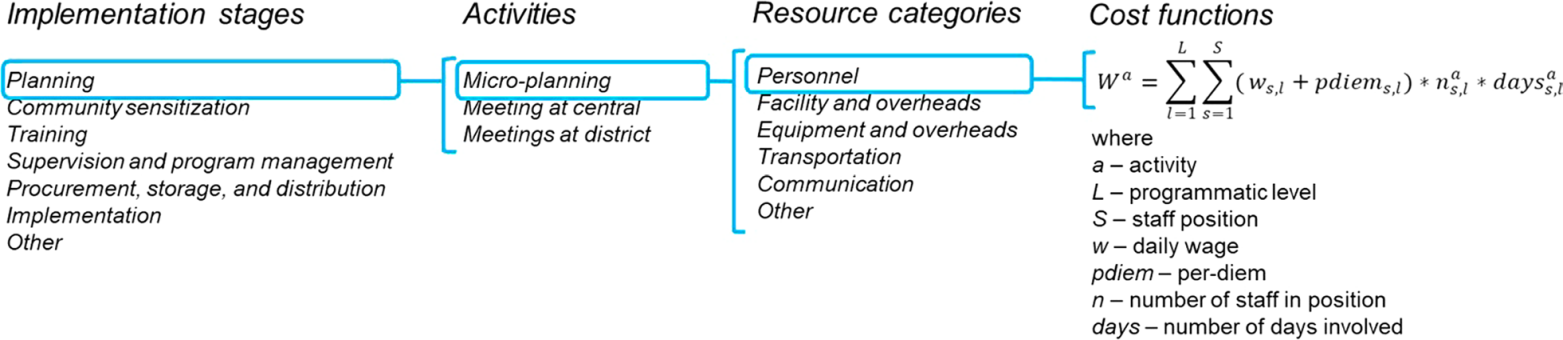
Schematic illustration of components of the micro-costing methodology. The figure illustrates the micro-costing approach by zooming in on the planning stage of implementation. For all interventions modelled here operational activities supporting planning covered micro-planning, meetings at central level, and meetings at district level. The chart details resource categories into which resource line items were grouped for purposes of evaluation and reporting; consistent resource groupings were adopted for all operational activities. The last column of the chart shows how micro-inputs (i.e. daily wages of programme staff, number staff days, number of staff) are combined within a cost-function to estimate cost of personnel supporting micro-planning. Similar cost-functions were defined for all other relevant resource categories within each operational activity along the intervention implementation cycle

The micro-costing methodology [23] is illustrated in Fig. 1. Capitalizing on the high resolution of data in the costing studies and operational documents from the trial, cost functions were defined at a low level of aggregation. Within each activity, costs were evaluated by resource category with economic valuation formalized in an equation. These represent largely linear combinations of prices and quantities summed over the corresponding staff or resource units, programmatic levels, and implementation stages. Capital items were annualized, and, also discounted, when reporting economic costs. The start-up costs related to planning, sensitization, and training activities supporting introduction of new interventions were treated as capital items, with utility life-years (ULY) top-coded to the number of years the intervention is implemented. Shared resources and overheads were valued based on use (i.e. number of days). Models were implemented and evaluated in Stata 14 SE [24].

For each intervention, the costing model formalized how resource use and service outputs (i.e*.* number of households sprayed per spray team per day) related to the setting. These relationships were quantified with data from the trials and supported with expert opinion of the implementing partner where data were lacking. Capacity constraints at higher programmatic levels were reflected in the number of days dedicated to sensitization, training, and supervision activities. In RR model, setting and health infrastructure were incorporated with parameters describing the number of health facilities per population target. For RACD the number of index cases followed-up was modelled as a function of *Pf*PR, health seeking for malaria, number of health facilities, number of CHWs and the capacity of CHWs to follow-up cases. For MDA these features determined duration of the campaign; it was assumed that CHWs could be recruited from nearby villages to support drug distribution as needed. For IRS the number of spray operators was modelled as a function of population size of the targeted area, target coverage (requiring information on the number of people per structure), and the number of structures that could be sprayed per operator per day.

The costing models were populated with quantities of resources that varied by intervention, and prices that were fixed within the setting. The prior were sourced directly from the reference implementation scenario, while the latter were obtained from the trials, published studies, President’s Malaria Initiative (PMI) country reports, and global cost depositories including the WHO-CHOICE and the country Multi-Year Plans for Immunization. Data sources by intervention are reported in Additional file 1: Table S1.

### Sensitivity analysis

Several strategies were pursued to assess generalizability of the cost estimates and the underlying cost model assumptions. First, a value range was defined for each model input and 500 sets of vectors of model parameters were simultaneously drawn from within that range, assuming uniform distribution, using Latin Hypercube sampling. These sets were re-sampled 10,000 times. Parameter ranges were sourced from the trial and the literature for all inputs characterizing the intervention, while a generic range between 50 and 200% of the reference parameter value was used to introduce variability in inputs describing programme overheads. Inputs of the costing model that were plausibly correlated were grouped together. One variable—“a multiplier”—was sampled for the group; all inputs were then adjusted by the same sampled value within the draw. Intervention costs were re-estimated with these sampled values.

Using the simulated data, the relative contribution of model inputs was evaluated by regressing the unit cost on inputs of the costing model and calculating the ratio of the variation explained (sum of squares) by the respective parameter as a fraction of total variation explained by the model. Since different parameterizations of costing models are possible, inputs of the costing model were grouped into five categories and the joint contribution of all inputs within the category was reported. Specifically, inputs that describe operational details of the implementation process were grouped under “intervention” category (1); inputs that characterize the health systems, parasite prevalence, and geography under “setting” (2); number of regions, districts, and HFCA population size under “scale” (3); prices of commodities, wages of programme staff under “price” (4), and inputs related to the economic valuation of resource use (i.e. discount rate and ULY assumptions) under “methods” (5). The list of model inputs by category is reported in Additional file 1: Table S2.

To highlight the contribution of individual parameters one-way sensitivity analyses were also implemented. Here, unit cost estimates were derived by setting one parameter at a time to the lowest and highest values of the corresponding range while keeping other inputs at reference values.

Finally, the cost implications of systematic correlations between the context and implementation of interventions at varying scales were explored using scenario analyses. The context was categorized by geographic accessibility and health system’s capacity. Scenarios modelled then hypothesized how implementation of interventions and the operational constraints due to features of the setting might change from the reference. Cost trajectories were obtained by smoothing point estimates generated by recalculating costs over different scales of implementation (i.e. allowing the number of regions, districts, and the size of HFCAs to vary) with lowess regression [25].

## Results

### Reference implementation scenarios

Table 1 presents key operational assumptions highlighting resource use at service delivery where implementation varies most between settings. Full implementation scenarios are shared in Additional file 2.

**Table 1:** Reference implementation scenarios by intervention

	Rapid reporting	Reactive case detection	Mass Drug Administration	Indoor residual spraying
Definition	Localized rapid reporting system of malaria diagnosed and treated cases and related commodities	Reactive focal testing and treatment of individuals living near clinical cases diagnosed and treated passively at health facilities or in community	Mass drug administration in a defined area without previous testing	Spraying interior surfaces of dwellings in a defined area with a residual insecticide
Scale	1 region, 3 districts, 20 HFCA each, 6000 population per HFCA	1 region, 3 districts, 20 HFCA each, 6000 population per HFCA	1 region, 3 districts, 20 HFCA each, 6000 population per HFCA	1 region, 3 districts, 50% of district HFCA targeted, 6000 population per HFCA
Level	HFCA	HFCA	HFCA	District
Staff	1 nurse/HF	8 CHW/HFCA	8 CHW/HFCA	42 operators/district
Operational details	2 days of training 1 nurse 0.5 days/ month collating entries and reporting Mobile phone and data Supervised by district and regional staff—DHIS2 malaria module, 20% of server, server maintenance fees, and IT support allocated to malaria reporting^1^	4 days of training 1 CHW 1 day to follow-up an index case 5 person radius around an index case Bicycle 1 CHW per HFCA receives a mobile phone and data Supervised by HF nurses, district and regional staff Existing DHIS2, 6% of DHIS2 running costs allocated to reporting for RACD^2^	4 days of training 1 adherence officer per 2 CHWs 75 persons reached per pair per day Supervised by HF nurses, district, regional and central staff NMCP vehicles and drivers used for distribution and supervision Length of campaign is 10 days^3^ 2 rounds per year	7 days of training 6 spray operators, 1 team leader per pair, 8 pairs per district 60 structures sprayed per day by team 5 people per structure Supervised by HF nurses, district, regional and central staff NMCP vehicles and drivers used for distribution and supervision Length of campaign is 29 days^4^ 1 round per year
Commodities		RDT, ALU	DHAP	*Actellic* 300 SC
Coverage	100%	100% of index cases; up to 5 index cases per CHW per week	85%	90% of targeted areas
Time valuation	Wages allocated based on time supporting rapid reporting Reporting nurse receives a monthly incentive for complete reporting	Economic value of time 0.36 USD/ day 1 CHW per HFCA collates and reports cases in the community and receive a monthly incentive for complete reporting	Economic value of time 0.36 USD/ day CHWs receive daily food allowances and an incentive award at the end of each MDA round	Spray operators receive wages, per-diems, and daily food allowances 20% receive a travel and lodging allowance to cover hard to reach areas

*HF* Health Facility, *HFCA* Health Facility Catchment Area, *CHW* Community Health Worker, *RDT* rapid diagnostic test, *ALU* artemether–lumefantrine

^1^DHIS2 infrastructure was allocated to RR assuming 20% of health facility visits are malaria-related

^2^DHIS2 running costs were allocated to RACD as a fraction of RR costs calculated as a ratio of the number of people tested in the community during RACD activities to the total number of people tested at health facilities in Zambia trial (MACEPA reporting)

^3^Length of MDA campaign was fixed at 10 days aligned with WHO recommendation [41]

^4^Length of IRS campaign was calculated based on the recommended number of spray operators, size of the district, and number of strictures sprayed per operator per day [42]

Malaria RR, as modelled here, relies on nurses to collate and report monthly information on diagnosis and treatment of malaria cases at health facilities and community. It was assumed that introduction of RR will not change reporting by CHWs that already routinely document on paper information on treatment and diagnosis in the community. To the extent that the introduction of RR in a given setting alters the workload of the community cadre (i.e. with training) or requires other additional resources (i.e*.* mobile phones), the corresponding inputs of the costing model would need to be updated to adequately capture these additional costs. Another important assumption is a pre-existing DHIS2 system to which a malaria module is added to support RR. Costs related to server purchase and maintenance overheads account for a significant fraction of the intervention costs, thus assumptions on how these are shared between the programme and reporting for other diseases will have important implications for costs. Here, server costs and other shared resources were allocated to RR assuming that about 20% of health facility visits in low-endemicity settings are malaria related.

Reference RACD implementation relies on an existing network of CHWs to follow up malaria cases. RACD-related reporting is assumed to be integrated within the existing surveillance infrastructure. The costing model accommodates the effectiveness cascade of RACD by allowing parameters related to access to treatment following an infection, fraction of index cases followed up, proportion of residents in eligible households present for testing and treatment during an RACD visit, as well as *Pf*PR to be varied [15]. At reference *Pf*PR and health-seeking rates, an average of four cases per week per HFCA would be counted in the model. This aligns well with the capacity for follow-up by CHWs suggested by the malaria elimination guidelines and the experience of the Zambia programme (5–10 cases per month can be followed up per CHW) [7, 26].

For MDA trade-offs between the number of CHWs mobilized for the campaign, the number of people that can be reached by a CHW per day, and the length of the campaign will determine the cost of the programme. Following normative guidance, it was assumed that drugs were distributed within 10 days in a given target area and that CHWs from neighbouring villages were recruited where capacity was insufficient to cover the area in 10 days. CHWs were assumed to receive a daily food allowance and an incentive at the end of each campaign round. This is similar to how MDA was programmatically implemented for control of soil-transmitted helminths infections and lymphatic filariasis [27, 28].

Unlike MDA that relies on community resources, IRS is a district level intervention. Spray teams move within the district from one targeted area to the next with the number of teams and the number of spray operators assigned based on the scale of implementation. Length of an IRS campaign is then determined by the number of spray operators recruited, the number of houses sprayed per day, and the population size. To reflect targeting of IRS at areas prone to outbreaks or identified as transmission sources the reference scenario assumed deployment in 50% of HFCAs. As transmission is further reduced across eliminating countries implementation of IRS might be shifted further down to HFCA level [29]. This would change the level and the distribution of costs and could be further explored with the costing model.

### Costing models

A summary of programmatic activities and key resource line items costed are reported in Additional file 1: Table S3, and fully in Additional file 2. Operational details of the reference scenario, described above, along with other setting and economic inputs were collated into an Excel dataset (Additional file 3). The costing models coded in Stata (Additional file 4) source this dataset to produce estimates of intervention costs under the reference implementation. To facilitate adoption of these cost models in optimization studies, these were further summarized in R functions that can be linked directly with epidemiological models (Additional file 1: Tables S4–S7). The functions yield an estimate of total intervention cost corresponding to the reference implementation. The analysts can either work with the full costing model or update cost components within the R function (a narrow menu of inputs) to propagate uncertainty in costs.

### Intervention costs

Under the reference implementation average annual cost across the four interventions ranged from about 0.12 USD for RR to 4.63 USD for two annual rounds of MDA per capita (Table 2, cost summaries per service output are reported in Additional file 1: Table S8). The difference between financial and economic cost highlights the contribution of health infrastructure including community resources. For RACD and MDA, where the operational model, consistent with programmatic experience from the region, assumed CHWs were incentivized for delivering these interventions, but were not directly compensated with either a per-diem or a wage, financial payments fail to adequately reflect the economic value of CHW time. Costs are higher in the first year reflecting expenditures related to initial start-up activities [i.e*.* micro-planning, sensitization, and training (Additional file 1: Tables S9, S10)]. The difference between the average annual cost and the first-year cost gives some indication of the penalty for switching between strategies.

**Table 2:** Average annual financial and *economic cost* per capita by intervention: reference implementation (USD, 2014)

Number of years	Financial cost				Economic cost			
	RR	RACD	MDA	IRS^a^	RR	RACD	MDA	IRS^a^
1	0.19	1.07	4.00	1.71	0.27	1.27	4.63	2.06
5	0.15	0.65	3.72	1.57	0.22	0.75	4.28	1.86

Intervention costs per capita (total population) reflect reference implementation presented in Table 1 above and Additional file 2. Estimates in the first-row show costs incurred in the first year (i.e. the year the intervention is first introduced), assuming the intervention is only to be deployed for 1 year. The second row gives the average annual economic cost assuming each intervention is implemented annually for 5 years

^a^In the reference implementation 50% of structures/population targeted by IRS (denominator for the unit cost is total population). Equivalent cost summaries per output are reported in Additional file 1: Table S7. Costs by implementation stage and cost structure are reported in Additional file 1: Tables S9, S10

Intervention costs per capita (total population) reflect reference implementation presented in Table 1 and Additional file 2. Estimates in the first-row show costs incurred in the first year (i.e*.* the year the intervention is first introduced), assuming the intervention is only to be deployed for 1 year. The second row gives the average annual economic cost assuming each intervention is implemented annually for 5 years. Note that in the reference implementation only 50% of structures/ population are targeted by IRS (denominator for the unit cost is total population). Equivalent cost summaries per output are reported in Additional file 1: Table S7. Costs by implementation stage and cost structure are reported in Additional file 1: Tables S9, S10.

The models can also be applied to explore costs of intervention packages. Introducing the four interventions at reference implementation will add up to 8.1 USD per capita in the first year, nearly four times the current spending on malaria in high burden countries, and two to four times above the current malaria spending in E8 countries [1]. IRS and MDA—the two “accelerator” interventions—make the largest contribution to total cost of the strategy accounting for 25% and 57% of total expenditure in the first year. Scaling down MDA in subsequent years to half of HFCAs will lower cost of the package to 5.9 USD per capita, further reducing IRS to 25% of HFCA areas will decrease cost of the strategy to 5 USD. This translates to savings of over a million USD compared to the reference package.

Models can help quantify possible cost savings from co-introduction or co-deployment of interventions. For instance, simultaneous introduction of RACD and MDA might be possible, since both rely on the CHW delivery platform. Suppose that when RACD and MDA are co-deployed planning, sensitization, and training activities could be shared thus lowering cost of the reference strategy to 7.5 USD per capita in the first year.

### Sensitivity analysis

Moving away from the reference implementation, variation in costs of the four interventions was assessed by sampling model inputs; thus, relaxing all parameter assumptions, and evaluating the resulting cost distributions (Additional file 1: Figures S1, S2). Reference costs were found to be on the lower side for all four interventions: the distribution mode of average annual economic cost per capita over 5 years was estimated at 0.21, 1.17, 6.42, and 2.79 USD for RR, RACD, MDA, and IRS, respectively (compare these to Table 2 estimates). Comparing reference input values against the parameter range characterizes the reference implementation modelled as a well-resourced setting with high operational efficiency (Fig. 3). Across the four interventions, assumptions on key operational inputs and health systems capacity are at the higher end of the range, corresponding to a lower unit cost.

**Fig. 2:**
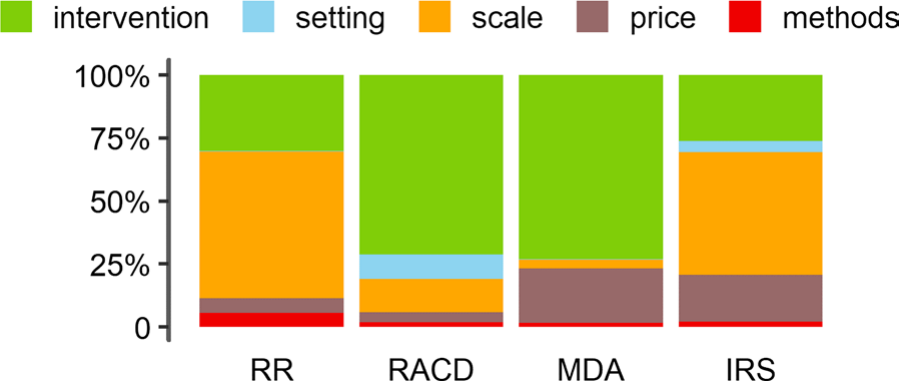
Bootstrap analysis of average annual *economic cost* per capita: unit cost (USD, 2014) and relative contribution of inputs by category. Colour segments of the stacked bars above correspond to the relative joint contribution of model inputs grouped into either of the five categories, describing intervention (green), setting (blue), scale (orange), price level (brown), and methods (red) to intervention unit cost. Proportions represent the joint contribution of model inputs within each category as a fraction of total variation in average annual economic cost per capita explained by the model. These were obtained by regressing cost per capita on model inputs sampled from 500 model parameter sets simultaneously drawn 10,000 times from a uniform distribution within the corresponding parameter range (Additional file 3). Model inputs by category are listed in Additional file 1: Table S2. Equivalent distributions for cost per outputs are shown in Additional file 1: Figure S1. *RR* Rapid Reporting, *RACD* reactive case detection, *MDA* Mass Drug Administration, *IRS* indoor residual spraying

**Fig. 3:**
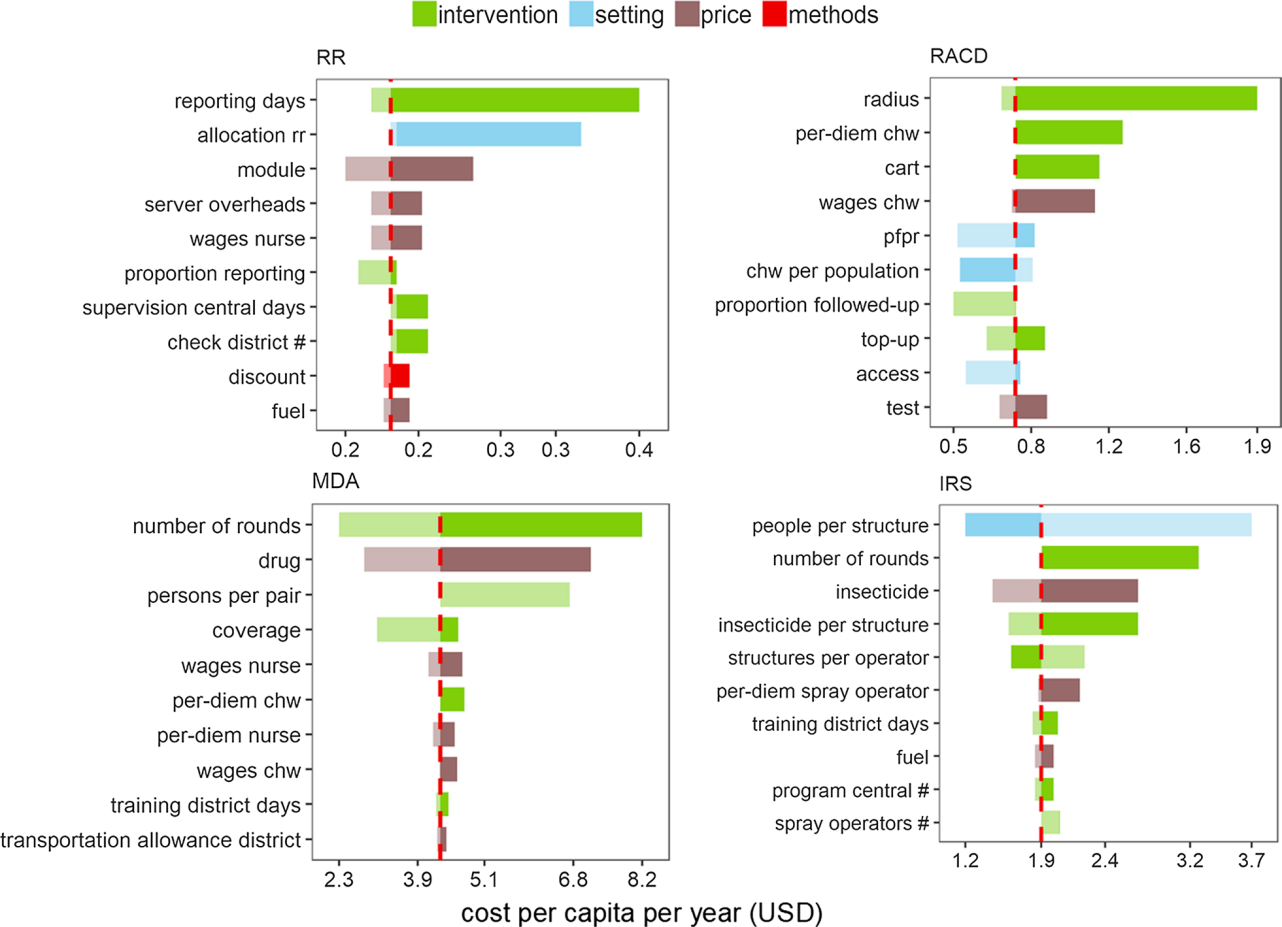
One-way sensitivity analysis of average annual *economic cost* per capita (USD, 2014) at reference implementation. Tornado plots show top 10 model inputs with the highest impact on intervention unit cost when varied over its’ minimum and maximum while keeping all other inputs at reference values (Additional file 1: Table S11). Bar lengths indicate the value of unit cost at highest—darker shade, and lowest—lighter shade, value of the respective parameter. Bar colour highlights input category. Red dashed lines give the reference estimate. Inputs describing scale of implementation (number of people reached) dominate the unit cost defined in terms of cost per capita; tabulations are thus shown only for parameters related to intervention (green), setting (blue), price (brown), and methods (red). Equivalent tabulations for cost per output are presented in Additional file 1: Table S2. Impact of scale parameters on estimated unit costs is explored in Fig. 4, and Additional file 1: Fig. S3. Reference implementation detailed in Table 1, further details in Additional file 1: Table S3 and Additional file 2. *RR* rapid reporting, *RACD* reactive case detection, *MDA* Mass Drug Administration, *IRS* indoor residual spraying

In Fig. 2 and Additional file 1: Figure S3, these sampled cost distributions were decomposed into relative contributions of model inputs aggregated into five core categories. One-way sensitivity analyses highlight inputs within each parameter category that have the highest impact on cost per capita (Fig. 3; Additional file 1: Figure S4). The relative importance of individual parameters depends on the selected range over which these were varied and the model structure. Parameter ranges chosen were loosely informed by the literature. These tabulations are presented here as a further validation of the model: given a plausible range of input values, model outputs are consistent with current understandings of cost drivers of these programmes.

Overall, parameters related to intervention design, service output and use of resources at delivery explain most of the variation in cost per capita for all interventions except RR and IRS. For RR and IRS, scale is the dominant driver due to the large initial investment needed to introduce these interventions. It covers spray pumps and spray operator kits for IRS and server and module development for RR as well as planning, training, and sensitization in the first year. Intervention-related inputs describing the volume of insecticide per structure and the number of structures sprayed per operator per day produce large gradients in unit cost of IRS; increasing the volume per m2 from 200 to 600 cm3 increases unit cost from 1.9 USD to nearly 3 USD per capita in the reference implementation. For RR—increasing the time for malaria reporting to 4 days per month (as seen in Ethiopia MACEPA pilot) instead of a quarter of a day in the reference implementation nearly doubles the intervention cost. For RACD costs, the target search radius and per-diems paid to CHWs dominate intervention parameters. Increasing the search radius to 30 people, as seen in the Senegal pilot, and some of the HFCAs in Zambia, will increase cost of RACD to nearly 2 USD per capita. For MDA the number of rounds per year and the number of people treated per CHW per day have the largest impact on cost when varied singly. Parameters characterizing the setting including the number of health facilities and number of CHWs per population target, access to treatment, and distances between programmatic levels are important for RACD and IRS. The number of people per structure—another setting parameter—modulates translation of population to structures targeted for IRS, which in turn determines requirements for spray operators and insecticide in the cost model. Price is a key driver for interventions with a high fraction of costs attributable to commodities such as MDA.

**Fig. 4:**
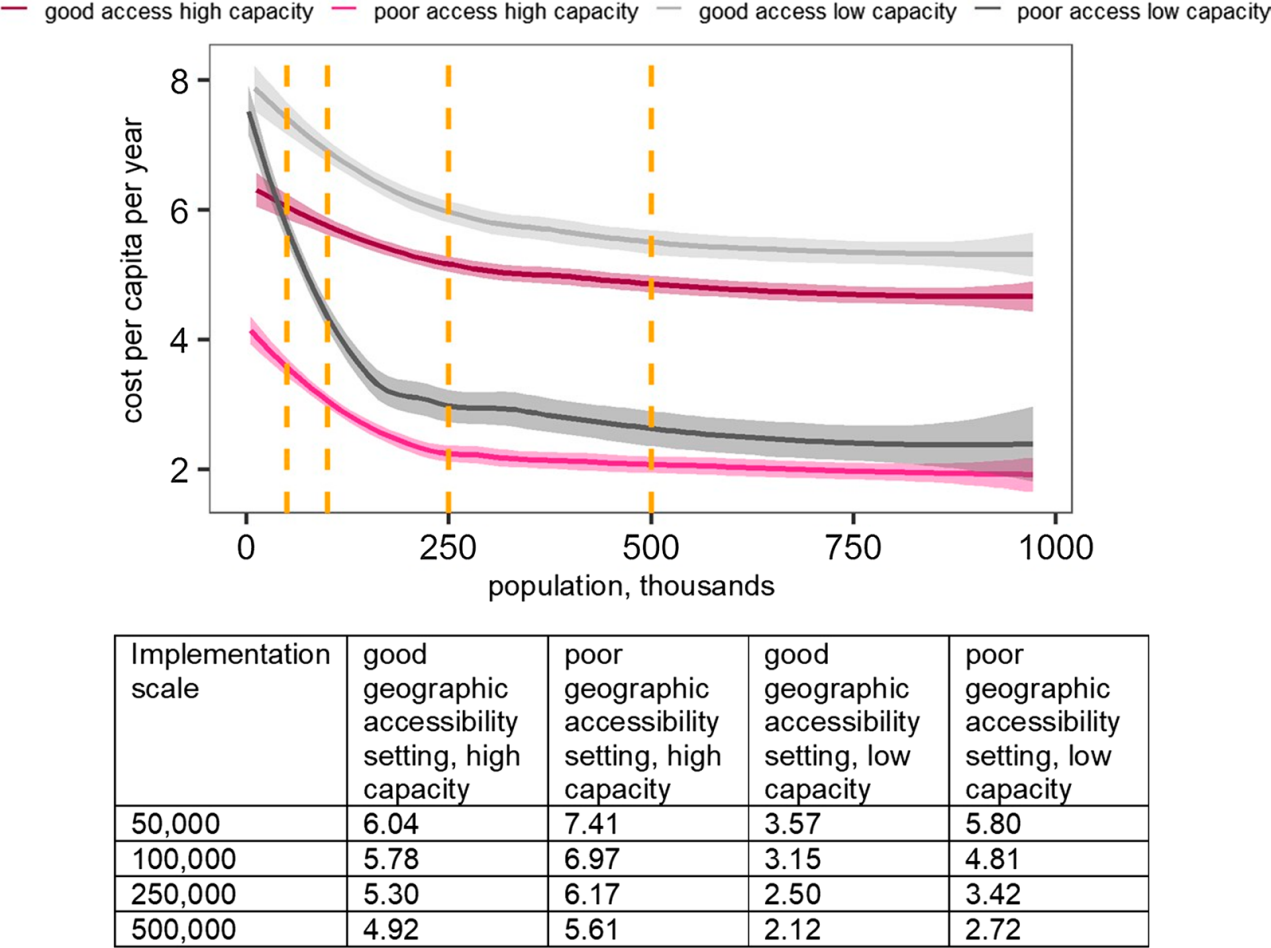
Mass Drug Administration cost per capita per year by setting and scale (USD, 2014). Each curve represents the intervention cost trajectory for the four setting types obtained by fitting a Loess curve to cost estimates modelled at various implementation scales. Shaded areas around the curves illustrate variation in the cost estimate due to different ways in which a given implementation scale can be achieved: by increasing the population size of the HFCA, increasing the number of HFCAs, or increasing the number of districts or regions where the intervention is deployed. Setting types are described in Additional file 1: Table S12. Equivalent figures for other interventions are shown in Additional file 1: Figure S5

Cost implications arising from systematic correlations between model inputs within a setting were explored using scenario analyses. Specifically, of interest here is the magnitude of cost gradients resulting from interactions between contextual features and implementation of interventions. For the four setting types, characterized by geographic accessibility and resource capacity, explicit assumptions were made on how implementation of interventions and key operational inputs might vary in response to these features (Additional file 1: Table S12). MDA coverage was assumed to decrease from 90 to 50% and the annual number of rounds to decrease from two to one in low compared to high capacity setting; the number of people treated per day to decrease from 75 to 50 in low compared to high geographic accessibility setting. Figure 4 illustrates how these, and other correlations detailed in Additional file 1, modify the relationship between MDA cost and scale.

Scenarios with lower efficiency are more sensitive to scale—notice steeper slopes at lower scales for the two poor accessibility settings. Capacity is dominated by scale and other parameters at higher population targets showing convergence of costs for the two low capacity scenarios. When varied singly at a reference scale, these parameters result in a range between 2.3 and 4.7 USD per capita per year compared to the reference estimate of 4.2 USD. Allowing implementation to vary by setting yields a range between 3.6 and 7.4 USD per capita if deployed in a population of 50,000, 3.2 and 7 USD per capita in a population of 100,000, and 2.1 and 5.6 USD per capita in a population of 500,000. Population size targets between 50,000 and 100,000 are in between district and region levels. Variation in costs is even greater at lower population scales between 2000 and 10,000 people representative of HFCA.

Economies of scale are evident in cost trajectories of all interventions up to population size of 200,000 (See Additional file 1: Figure S5). The relative importance of scale for different interventions is consistent with cost summaries in Additional file 1: Tables S9, S10 [note the share fixed costs (i.e*.* related to start-up activities and capital items)]. The figure demonstrates that differences in cost of interventions between settings are greatest at lower implementation scales, which further implies that the relative ranking of interventions may differ depending on the operational level at which strategies are defined.

## Discussion

This paper proposes models that rely on data from pilot studies and secondary sources to derive an estimate of intervention costs to inform introduction of new tools and optimization of malaria intervention strategies. Grounded in operational insight, the models produce locally relevant estimates of costs and should enable programmes to explore potential efficiency gains and cost savings from alternate delivery strategies. By formalizing the translation of operational data into an estimate of intervention costs, these models overcome one of the important challenges in standardizing economic evaluation of malaria programmes.

### From pilot implementation to programmatic delivery

Cost data are increasingly collected along field trials to inform economic evaluation of new health interventions. While efficient, the short-comings of this strategy often restrict extrapolation beyond pilots and thus limit relevance of the evidence for policy guidance [30]. In the costing studies conducted along MACEPA pilots that were guided by a well-defined methodology [13], variation in *choices* analysts made with respect to the scope of resources to be costed, *assumptions* on utility life-years of sensitization and training, and time at delivery were the primary sources of differences in cost of interventions between sites. Analysts relied on own judgement to adjust for co-deployment of interventions within the pilots and to disentangle resources used for research from implementation activities. For instance, in MACEPA pilots diagnostic testing to inform epidemiological outcomes was conducted alongside drug distribution, requiring additional community workers and increasing the length of household visits and length of the campaign, compared to what would have been required to support drug distribution within an MDA campaign. When the models were applied to derive intervention costs from the trial data, thus adjusting for these inconsistencies, estimates differed by an order of magnitude from those reported in the pilot studies (Additional file 1: Table S14). Finally, isolating resource use attributable to partner contribution for trial management and oversight, supervision, technical support, etc. was both challenging and raised further questions about local capacity to support these functions and about implications for effectiveness of interventions outside of the trial. Updating resource use assumptions on these inputs with values representative of experience of programmes in endemic African countries resulted in about 20% lower cost across the four interventions (Additional file 1: Table S14).

### Intervention cost drivers

The operational inputs were shown to be important drivers of costs and key to understanding trade-offs between interventions. While limitations of the trial data precluded empirical evaluation of the relationship between service output and context, these dimensions were incorporated into our costing models by explicitly defining a level of health infrastructure for the setting and drawing on expert opinion of in-country partners and secondary data sources to inform the translation. The strategy broadly aligns with earlier work within the WHO-CHOICE project that sought to incorporate health systems into optimization frameworks [31, 32]. The scenario analyses highlighted how the cost implications of intervention design might be magnified by setting specific features.

The value of trial data for programmatic decisions could be further strengthened by designing data collection to understand variability in resource use with respect to the setting and aspects of intervention design. For IRS, important quantities relate to the number of people protected/ structures sprayed per day and the volume of insecticide. PMI already routinely collects information on these parameters [33]; what is missing, however, is the link between these quantities and the broader context of the programme. For interventions relying on community volunteers, understanding the importance of incentives paid in the trials and contribution of supportive supervision for service outputs are key when evaluating delivery at scale. Compensation of community cadre varies greatly across the region from a volunteer basis with some incentives and per-diems for training (i.e. Zambia) to a paid Ministry of Health staff position integrated within the formal health sector (i.e. Senegal, Ethiopia) [34]. During the early stages of RACD introduction in Southern Province in Zambia, less than a quarter of eligible index cases were followed by CHWs with human resource constraints cited as the primary reason for low follow-up [14]. Thus, when evaluating malaria elimination strategies demands on CHW time need to be considered [35].

Commodities are key cost drivers for RACD (at assumed prevalence and positivity rate), MDA, and IRS (Additional file 1: Tables S9, S10). Second to commodities, costs incurred at point of delivery (i.e. at drug distribution or house spraying) are the next most important driver. These costs primarily cover compensation of field workers which are in turn a function of length of the campaign and other features related to intervention design and the setting (including age target, population density, number of houses that can be sprayed per operator per day). For RR, that requires an initial investment to acquire the server, the bulk of costs is driven by programme support and supervision. A key challenge for RR, but equally for other interventions, is poor evidence base on how these and other supportive activities modify resource requirements and impact on intervention effective coverage and cost.

### Using the models to derive an estimate of intervention costs

In this paper, the models were applied to extrapolate from MACEPA trials to a generic setting, yielding cost estimates that broadly align with the literature (Additional file 1: Table S15). In the same manner as the authors proceeded here, by critically evaluating normative guidance on implementation of interventions against the operational data from the trials, analysts and programme managers could update the respective inputs of the models to contextualize further interventions modelled to derive setting specific costs.

The detailed enumeration of resources and operational activities supported by the models ensures that every aspect of intervention, as it is implemented in a specific setting, can be adequately represented and costed. This flexibility is the key strength of the approach detailed here, it comes, however, at a price—the extensive data requirements to populate the model. The paper showed how trial and secondary data could be triangulated to source these data. The low level of aggregation within the models supports transferability of data across studies within a setting (i.e*.* wages of nurses are the same within a setting regardless of the intervention they deliver). Curated databases of prices for an extensive menu of micro-inputs [19, 36], developed recently to strengthen the evidence base for economic evaluation in LMICs, make the strategy presented viable for future prospective evaluation of new interventions.

### Using the models to inform resource allocation

Modelling and simulation of infectious disease dynamics are increasingly applied to guide thinking on optimal intervention strategies (including use of new tools) to achieve burden reduction or elimination [37]. By linking-in costs, such modelling allows decision makers to evaluate trade-offs between different strategies on the basis of costs and benefits and informs optimal allocation across interventions within the available budget envelop. The costing models presented here facilitate alignment of assumptions between epidemiological and economic inputs. Importantly, by fixing the scope of the evaluation, harmonizing assumptions on resource use, setting-specific inputs, and through a consistent application of economic valuation methods the models enable unbiased comparison between interventions.

Operational details of interventions, scale of implementation and capacity were all shown to have important implications for costs and effectiveness of elimination strategies. Operational scenarios presented and, more formally the cost models, relate these features to service outputs thus supporting use of modelling to optimize implementation of interventions. Work by Gao and colleagues is an example of a modelling study that explicitly incorporated setting, operational and logistic constraints on implementation of MDA to inform design of an elimination strategy. The authors demonstrated that shorter campaigns enabled by larger drug distribution teams would increase the likelihood of elimination in areas with true *Pf*PR under 3% and where population is highly mobile; in more static populations, deploying smaller teams would be cost optimal and as impactful. Systems capacity can be further incorporated in impact models by limiting the level to which interventions can be scaled-up, varying time over which target coverages are reached, and through scenario analyses where coverages and effectiveness of interventions are varied depending on the context [38].

In low-endemicity settings, in particular, due to reactive nature of programmatic response, the spatial and temporal pattern of outbreaks, the health infrastructure, and the capacity of the surveillance systems to adequately identify outbreaks need to be explicitly considered by economic and impact models aiming to inform policy decisions [39]. This further suggests the need to accommodate dependencies between the surveillance interventions such as RR and RACD and the effectiveness of targeted strategies and reactive responses and the underlying case management. A recent modelling study that explicitly considered these dependencies showed that although RACD may bring qualitative benefits in low-endemicity settings, improving case management may be more impactful [40].

## Conclusion

This paper illustrated the utility of costing models to synthesize data from pilot studies and secondary sources to inform evaluation of tools and optimization of intervention strategies by programmes. Grounded in operational insight, the models produce locally relevant estimates of intervention costs and allow programmes to explore potential efficiency gains and cost savings from alternate delivery strategies and intervention mixes. An important innovation of the models presented here is the explicit link between service outputs (i.e. effective coverage) and the health infrastructure. The value of this approach for decision-making is enhanced when primary cost data collection is designed to enable analysis of the efficiency of operational inputs in relation to features of the trial or the setting, thus facilitating transferability.

## Supplementary information

**Additional file 1.** A additional file containing additional figures and tables supporting interpretation of the analyses presented in the manuscript.

**Additional file 2.** Reference implementation scenarios detailing operational assumptions by intervention. A large Excel table that details by intervention resource lists and quantities of resources costed for each operational activity across all implementation stages (i.e. from the initial micro-planning on the new intervention to evaluation). The table offers a descriptive intervention narrative to aid in interpretation of the cost estimates derived here.

**Additional file 3.** Costing model inputs database. An Excel table that contains resource lists, respective prices and quantities for each intervention (tabs RR to IRS) that serve as inputs to the costing models (Stata do-files) shared in Additional file 3. For each micro-input the table gives the reference value associated with the reference implementation scenario, minimum and maximum value of the parameter, a flag that denotes how the parameter was treated in sensitivity analysis (i.e. varied within the range or according to the multiplier), resource category grouping matching Fig. 2, parameter label, units, description, and source of data. Additionally, tab “sensitivity” gives multiplier range for each parameter and grouping. Tab “archetypes” gives values of operational and setting inputs by setting archetype.

**Additional file 4.** Costing models by intervention and scope. The folder contains Stata do-files that detail how operational details as per Additional file 1, are combined with information on prices and quantities of resources collated in Additional file 2 to produce and estimate of intervention costs. The files can be run together with the datasets in Additional file 2 to reproduce estimates presented in the manuscript. The files can be used to also model alternative implementation of interventions or produce estimates for settings other than the reference.

## Data Availability

All data generated or analysed during this study are included in this published article and its additional information files.

## Abbreviations

MACEPA: Malaria Control and Elimination Partnership in Africa at PATH
CHWs: Community Health Workers
LMICs: Low and middle income countries
RR: Malaria rapid reporting
RACD: Reactive case detection
MDA: Mass Drug Administration
IRS: In-door residual spraying
DHIS2: District Health Information System 2
RDT: Rapid diagnostic test
WHO: World Health Organization
HFCA: Health Facility Catchment Area
*Pf*PR: *Plasmodium falciparum* parasite prevalence
ULY: Utility life-years
PMI: President’s Malaria Initiative
USD: United States Dollar
SMC: Seasonal malaria chemoprophylaxis
NTD: Neglected tropical diseases
