# Supplementary material for: Costing malaria interventions from pilots to elimination programmes
Source: Malar J. 2020 Sep 14;19:332. doi: 10.1186/s12936-020-03405-3 (PMC7491157; doi:10.1186/s12936-020-03405-3)
Supplement: Supplementary file 1 — Additional file 1. A additional file containing additional figures and tables supporting interpretation of the analyses presented in the manuscript. [file 12936_2020_3405_MOESM1_ESM.docx]

**Additional Information: Additional file 1**

**COSTING MALARIA INTERVENTIONS FROM PILOTS TO ELIMINATION PROGRAMS**

Galactionova K.^1,2^, Velarde M.^1,2^, Silumbe K.^3^, Miller J.^3^, McDonnell A.^4,5^, Aguas R. ^4,5^, Smith T.A.^1,2^, and Penny M.A.^1,2^

^1^ Dept. of Epidemiology and Public Health, Swiss Tropical and Public Health Institute, Basel, Switzerland ^2^ University of Basel, Basel, Switzerland

^3^ Malaria Control and Elimination Partnership in Africa, (MACEPA), PATH

^4^ Mahidol Oxford Tropical Medicine Research Unit, Faculty of Tropical Medicine, Mahidol University, Bangkok, Thailand

^5^ Centre for Tropical Medicine and Global Health, Nuffield Department of Medicine, University of Oxford, Oxford, United Kingdom

*Corresponding author:

Katya Galactionova

Swiss Tropical and Public Health Institute

Socinstrasse 57,

4051 Basel, Switzerland

e-mail: [e.galactionova@unibas.ch](mailto:e.galactionova@unibas.ch)

**Table S1. References supporting implementation scenarios by intervention**

| Intervention | Published studies | Normative guidelines |
| --- | --- | --- |
| Rapid Reporting | [1-4] | [5] |
| Reactive Case Detection | [6-10] | [11] |
| Mass Drug Administration | [12-20] | [21] |
| Indoor Residual Spraying | [22, 23] | [24] |

**Table S2. Parameters of the costing model by input category**

| Input category | Rapid Reporting | Reactive Case Detection | Mass Drug Administration | Indoor Residual Spraying |
| --- | --- | --- | --- | --- |
| Intervention | data use by dCHF nurses, days checking data by district staff, days planning meetings, days planning, days program management, days reporting by HF nurses, days supervision, days training, number district staff supporting audits at HF, number district staff checking data, number staff planning, number staff program management, number staff sensitization, number staff supervision, number staff training, proportion HF reporting, top-up paid to nurses for reporting, number of trainees per training session | data use by CHW, data use by HF nurses, days checking data by district and HF staff, days to follow-up an index case, days planning meetings, days planning, days program management, days reporting by HF nurses, days supervision, days training, food allowance, CHW kit, number district staff conducting data audit, number of district staff checking data, number of mobile phones per HFCA, number staff planning, number staff program management, number staff sensitization, number staff supervision, number staff training, number of vehicles for drug distribution, number of sensitization activities per district, number of CHWs per pair following-up index cases, per-diem to CHW, proportion of index cases followed-up, proportion of HF reporting, radius around index case, top-up paid to dCHW for reporting, number of trainees per training session | coverage, data use by HF nurses,  days planning meetings, days planning, days program management, days reporting by HF nurses during MDA campaign, days supervision, days training, CHW kit, days sensitization, number of persons treated per CHW pair per day, number of MDA CHW teams supervised by HF nurse, number staff planning, number staff program management, number staff sensitization, number staff training,  number of sensitization activities per district, number of CHWs per pair conducting MDA, per-diem to CHW, number of MDA rounds per year, incentive paid to CHW at the end of MDA round, number of trainees per training session | coverage, days planning meetings, days planning, days program management, days supervision, days training, structures sprayed per day per operator, spray operator kit, number of spray operators per team, number staff planning, number staff program management, number staff sensitization, number staff supervision, number spray operators per team, number staff training, number vehicles per district, number of sensitization activities per district, volume of insecticide per structure, number of IRS rounds per year, number of trainees per training session |
| Setting | health seeking for malaria, distances between program levels, number of CHW per population target, number of HF per population target, *Pf*PR, proportion allocation of shared resources to RR | health seeking for malaria, distances between program levels, number of CHW per population target, number of HF per population target, *Pf*PR, positivity rate around an index case, proportion allocation of shared resources to RR, proportion allocation of shared resources to RACD, proportion of population within the target radius available for fTAT | health seeking for malaria, distances between program levels, number of CHW per population target, number of HF per population target, *Pf*PR | health seeking for malaria, distances between program levels, number of CHW per population target, number of HF per population target, persons per structure, percent vehicles rented |
| Scale | number of districts, number regions, HFCA population size | number of districts, number regions, HFCA population size | number of districts, number regions, HFCA population size | number of districts, number regions, HFCA population size, proportion of HFCA’s targeted |
| Price | prices of equipment, vehicles, fuel, facility and vehicle rental, overheads, wages and per-diems of program staff, allowances, stationaries, consumables | prices of equipment, vehicles, fuel, facility and vehicle rental, overheads, wages and per-diems of program staff, gifts, drugs, diagnostics, allowances, stationaries, sensitization activities and materials, other print, consumables | prices of equipment, vehicles, fuel, facility and vehicle rental, overheads, wages and per-diems of program staff, gifts, drugs, diagnostics, allowances, stationaries, sensitization activities and materials, other print, consumables | prices of equipment, vehicles, fuel, facility and vehicle rental, overheads, wages and per-diems of program staff, gifts, insecticide, environmental compliance and waste management, allowances, stationaries, sensitization activities and materials, other print, consumables |
| Methods | ULY of all capital items (i.e. buildings, equipment, furniture, etc.), ULY of training and sensitization activities, discount rate | ULY of all capital items (i.e. buildings, equipment, furniture, etc.), ULY of training and sensitization activities, discount rate | ULY of all capital items (i.e. buildings, equipment, furniture, etc.), ULY of training and sensitization activities, discount rate | ULY of all capital items (i.e. buildings, equipment, furniture, etc.), ULY of training and sensitization activities, discount rate |

**Table S3. List of operational activities and key resource line items by intervention implementation stage**

| Implementation stage | Operational activities | Resource line items | Rule for allocation of shared resources to intervention |
| --- | --- | --- | --- |
| Planning | Micro-planning at central, regional, and district levels, planning meetings at central level and district levels | Wages and per-diems of program staff, transportation, equipment, facility and related overheads, supplies | Number of meetings, number of days |
| Procurement | Procurement | Commodities (i.e. drugs, RDTs, etc.) and related equipment, wastage, CHW/ spray operator kits | 100% |
| Storage | Storage of commodities and related equipment at central and district and/or HFCA levels | Facility and overheads, store-keeper | Volume, days of storage |
| Distribution | Distribution of commodities and related equipment from central to district/HFCA levels | Vehicles and overheads, fuel, driver wages and per-diems, program staff wages and per-diems, loaders/ off-loaders per-diems | Volume, distance |
| Training | Training of supervisors, trainers, implementation staff at central, regional/district and HFCA levels | Wages and per-diems of program staff, transportation, equipment, bus rental for piloting and field practice, facility and related overheads, commodities, supplies and consumables | Number of days |
| Community sensitization | Advocacy meetings with community members, community forum, community entry meetings at central and district levels, social mobilization at community level | Wages and per-diems of program staff, gifts and incentives to community members, print materials, transportation, equipment, facility rental (hall or tent), chair rental, sound equipment rental, drama group fees, supplies and consumables, public announcement system, radio broadcasts | Number of meetings, number of days |
| Program management and supervision | Program management at central, regional, district levels and supervision by central, regional, district, and HF staff, IT infrastructure (RR, RACD) | Wages and per-diems of program staff, transportation, equipment, facility and related overheads, supplies | Number of days, distance |
| Implementation | Service delivery in community, reporting at HF level (RACD, MDA), stock management (MDA, IRS), | Wages and per-diems of program staff, food allowance, lodging allowance, data, transportation, equipment, facility and related overheads, supplies | Number of HF (RR), number of index cases (up to capacity) (RACD), number of campaign days (MDA, IRS), % of households targeted (IRS), scale |
| Other intervention specific | Data audit (RR), routine data quality checks (RR, RACD), pharmacovigilance (MDA), inspection (IRS), environmental compliance (IRS), waste management (MDA, IRS), review meeting at central level (IRS) | Wages and per-diems of program staff, transportation, equipment, facility and related overheads, environmental compliance, waste management, supplies | Number of days, distance, volume |

RR= Rapid Reporting; RACD= Reactive Case Detection; MDA= Mass Drug Administration; IRS= Indoor Residual Spraying

**Table S4. Economic cost ingredients summaries of costing models for the reference implementation and R functions for linking with impact models: malaria rapid reporting**

| Activity | Parameter | Unit cost (first year/ thereafter) | Units | Impact model inputs for linking |
| --- | --- | --- | --- | --- |
| Planning | *p* | 0.0339 | per person per year | pop, yrs |
| Procurement and distribution of mobile phones and supplies | *k* | 0.0042 | per person per year | pop, yrs |
| Training | *t_1_/ t_2_* | 0.0293/  0.0079 | per person per year | pop, yrs |
| Program management, IT support, and supervision |  |  | per person per year | pop, yrs |
| - Server | *y* | 15000 | per year proportional to use for malaria RR | yrs, prop_rr |
| - Server overheads | *q* | 50000 | per year proportional to use for malaria RR | yrs, prop_rr |
| - Module | *l* | 114000 | per program |  |
| - Other program | *m* | 0.0122 | per person per year | pop, yrs |
| Implementation | *f* | 0.0538 | per person reporting per year | pop, prop_report, yrs |
| Other: data quality | *o* | 0.0256 | per person per year | pop, yrs |
| $TC=\frac{p+t_{1}}{a_{p}}{*uly}_{p}+\frac{t_{2}}{a_{t}}*\left( \left( yrs-1 \right)\div3 \right)+\left( k+m+f*prop_{report}+o \right)*yrs)*pop+\frac{l}{a_{l}}*{uly}_{l}+(y+q)*prop\_rr*yrs$  where  TC is total cost of malaria RR under reference implementation  a_p_, a_t_ and a_l_ are annualization factors for start-up activities and routine training (t, conducted every 3 years following introduction), and RR module  pop is total population in the area where RR program is implemented  prop_report is proportion on CHWs reporting  prop_rr is proportion of DHIS2 server and server overheads to be allocated to malaria RR  pos_rate is positivity rate around index case  yrs is number of years RACD program is implemented  uly_p_ is ULY for activities conducted in preparatory, start-up stage of implementation  uly_l_ is ULY for malaria RR module | | | | |
| **R function:** | | | | |
| calc_RR_Costs<- function(yrs,pop,prop_rr,prop_report,r) {  ingr<- list ("p"= 0.0339, "k"=0.0042, "t1"=0.0293, "t2"=0.0079, "y"=15000, "q"=50000,  "l"=114000, "m"=0.0122, "f"= 0.0538, "o"=0.0256)  uly_p<- if(yrs<5) yrs else 5  uly_t<- if (yrs<3) yrs else 3  uly_l<- if(yrs<10) yrs else 10  a_p<- ((1-(1+r)^(-uly_p))/r)  a_t<- ((1-(1+r)^(-uly_t))/r)  a_l<- ((1-(1+r)^(-uly_l))/r)  totalCost<- with(ingr, (((p+t1)/a_p)*uly_p+(t2/a_t)*((yrs-1)%/%3)+  (k+m+o+f*prop_report)*yrs)*pop +l/a_l*uly_l+(y+ q)*prop_rr*yrs)  return(totalCost)  }  calc_RR_Costs(5,360000,0.2000,1,0.03) | | | | |

**Table S5. Economic cost ingredients summaries of costing models for reference implementation and R functions for linking with impact models: malaria reactive case detection**

| Activity | Parameter | Unit cost (first year/ thereafter) | Units | Impact model inputs for linking |
| --- | --- | --- | --- | --- |
| Planning | *p* | 0.0573 | per person per year | pop, yrs |
| Procurement, storage, and distribution of commodities |  |  |  |  |
| - CHW kits and supplies (including mobile phones for dCHW) | *k* | 0.1001 | per person per year | pop, yrs |
| - RDTs | *w* | 1.5507 | per person tested around an index case per year | pop, n_index, prop_follow, radius, prop_tat, yrs |
| - Antimalarial drugs | *d* | 1.9851 | per positive case around an index case per year | pop, n_index, prop_follow, radius, prop_tat, pos_rate, yrs |
| Training | *t_1_/ t_2_* | 0.5375/ 0.1605 | per person per year | pop, yrs |
| Sensitization | *s* | 0.0788 | per person per year | pop, yrs |
| Program management and supervision | *m* | 0.0624 | per person per year | pop, yrs |
| Implementation^*^ | *f* | 5.4329 | per index case followed up per year | pop, n_index, prop_follow, yrs |
| Other: data quality | *o* | 0.0077 | per person per year | pop, yrs |
| $TC=\left( \frac{p+s+t_{1}}{a_{p}}*{uly}_{p}+\frac{t_{2}}{a_{t}}*\left( \left( yrs-1 \right)\div3 \right)+\left( k+m+o \right)*yrs \right)*pop+$  $(\left( \left( d*pos_{rate}+w \right)*prop_{tat}*radius+f \right)*n_{index}*prop_{followed})*yrs$  where  TC is total cost of RACD program under reference implementation  a_p_ and a_t_ are annualization factors for start-up activities and routine training (t, conducted every 3 years following introduction)  pop is total population in the area where RACD program is implemented  n_index is number of index cases per year  prop_follow is proportion on index cases followed-up by CHWs  radius is number of people around and index case tested during RACD  prop_tat is proportion of residents around index case available for testing and treatment  pos_rate is positivity rate around index case  yrs is number of years RACD program is implemented  uly_p_ is ULY for activities conducted in preparatory, start-up stage of implementation | | | | |
| **R function:** | | | | |
| calc_RACD_Costs<- function(yrs,pop,n_index,prop_follow,radius,prop_tat,pos_rate,r) {  ingr<- list ("p"= 0.0573, "k"=0.1001, "w"=1.5507, "d"= 1.9851, "t1"=0.5375, "t2"=0.1605,  "s"=0.0788, "m"=0.0624, "f"= 5.4329, "o"=0.0077)  uly_p<- if(yrs<5) yrs else 5  uly_t<- if(yrs<3) yrs else 3  a_p<- ((1-(1+r)^(-uly_p))/r)  a_t<- ((1-(1+r)^(-uly_t))/r)  totalCost<- with(ingr, ((p+s+t1)/a_p*uly_p+t2/a_t*((yrs-1)%/%3)+ (k+m+o)*yrs)*pop+  (((d*pos_rate+w)*radius*prop_tat+f)*n_index*prop_follow)*yrs)  return(totalCost)  }  calc_RACD_Costs(5,360000,11520,1,5,0.8,0.183333,0.03) | | | | |

**Table S6. Economic cost ingredients summaries of costing models for reference implementation and R functions for linking with impact models: mass drug administration**

| Activity | Parameter  (first year/ thereafter) | Unit cost (first year/ thereafter) | Units | Impact model inputs for linking |
| --- | --- | --- | --- | --- |
| Planning | *p_1_/ p_2_* | 0.0573/ 0.0093 | per person per year | pop, yrs |
| Procurement, storage and distribution of CHW kits and supplies |  |  |  |  |
| - CHW kits and supplies | *k* | 0.0694 | per person per year | pop, yrs |
| - Antimalarial drugs | *d* | 1.7549 | per person treated per round per year | pop, yrs, rnds, cov |
| Training | *t_1_/ t_2_* | 0.4991/ 0.1605 | per person per year | pop, yrs |
| Community sensitization | *s_1_/ s_2_* | 0.0983/ 0.0321 | per person per year | pop, yrs |
| Program management | *m* | 0.0061 | per person per year | pop, yrs |
| Supervision | *s* | 0.0891 | per person per round per year | pop, yrs, rnds |
| Implementation | *f* | 0.4343 | per person treated per round per year | pop, yrs, rnds, cov |
| $TC=\left( \frac{p_{1}+t_{1}{+s}_{1}}{a_{p}}*{uly}_{p}+\left( p_{2}+s_{2}+t_{2} \right)*\left( yrs-1 \right)+\left( k+m+\left( s+\left( d+f \right)*cov \right)*rnds \right)*yrs \right)*pop$  where  TC is total cost of MDA program under reference implementation  a_p_ and a_t_ are annualization factors for start-up activities  pop is total population in the area where MDA program is implemented  rnds is number of MDA rounds per year  cov is MDA coverage per round  yrs is number of years MDA program is run  uly_p_ is ULY for activities conducted in preparatory, start-up stage of implementation | | | | |
| **R function** | | | | |
| calc_MDA_Costs<- function(yrs,pop,rnds,cov,r) {  ingr<- list ("p1"= 0.0573, "p2"= 0.0093, "k"=0.0694, "d"= 1.7549, "t1"=0.4991, "t2"=0.1605,  "s1"=0.0983, "s2"=0.0321,"m"=0.0061, "s"=0.0891, "f"= 0.4343)  uly_p<- if(yrs<5) yrs else 5  a_p<- ((1-(1+r)^(-uly_p))/r)  totalCost<- with(ingr,((p1+t1+s1)/a_p*uly_p+(p2+s2+t2)*(yrs-1)+(k+m+(s+(d+f)*cov)*rnds)*yrs)  *pop)  return(totalCost)  }  calc_MDA_Costs(5,360000,2,0.85,0.03) | | | | |

**Table S7. Economic cost ingredients summaries of costing models for reference implementation and R functions for linking with impact models: in-door residual spraying**

|  | Parameter | Unit cost (first year/ thereafter) | Units | Impact model inputs for linking |
| --- | --- | --- | --- | --- |
| Planning | *p_1_/p_2_* | 0.1309/ 0.0071 | *per person per year* | *pop, yrs* |
| Procurement including storage and distribution |  |  |  |  |
| - Spray operator kit | *k* | 0.0929 | *per person targeted per year* | *pop, target, yrs* |
| - Spray pumps and supplies | *p* | 0.1637 | *per person targeted per year* | *pop, target, yrs* |
| - Insecticide (Actellic) | *c* | 1.8744 | *per person protected per round per year* | *pop, target, cov, rnds, yrs* |
| Training | *t_1_/t_2_* | 0.4722/ 0.3249 | *per person targeted per year* | *pop, target, yrs* |
| Community sensitization | *s_1_/s_2_* | 0.1033/ 0.0371 | *per person per year* | *pop, yrs* |
| Program management | *m* | 0.1072 | *per person per year* | *pop, yrs* |
| Supervision | *s* | 0.1934 | *per person targeted per round per year* | *pop, target, rnds, yrs* |
| Implementation | *f* | 0.8042 | *per person protected per round per year* | *pop, target, cov, rnds, yrs* |
| Other |  |  |  |  |
| - Environmental compliance | *e* | 0.0174 | *per person targeted per round per year* | *pop, target, rnds, yrs* |
| - Waste management | *w* | 0.0113 | *per person protected per round per year* | *pop, target, cov, rnds, yrs* |
| - Inspection | *i* | 0.0332 | *per person targeted per round per year* | *pop, target, rnds, yrs* |
| - Annual review | *v* | 0.038 | *per person targeted per year* | *pop, target, yrs* |
| $TC=\left( \frac{p_{1}{+s}_{1}}{a_{p}}*{uly}_{p}+\left( p_{2}+s_{2} \right)*\left( yrs-1 \right)+m*yrs +\left( \frac{t_{1}}{a_{p}}*{uly}_{p}+t_{2}*\left( yrs-1 \right)+(k+p+v+\left( e+i+s+\left( c+f+w)*cov \right)*rnds \right)*yrs \right)*target \right)*pop$  where  TC is total cost of MDA program under reference implementation  a_p_ and a_t_ are annualization factors for start-up activities  pop is total population in the area where IRS program is implemented  rnds is number of IRS rounds per year  cov is MDA coverage per round  yrs is number of years IRS program is implemented  uly_p_ is ULY for activities conducted in preparatory, start-up stage of implementation | | | | |
| **R function** | | | | |
| calc_IRS_Costs<- function(yrs,pop,target,rnds,cov,r) {  ingr<- list ("p1"= 0.1309, "p2"= 0.0071, "k"=0.0929, "p"= 0.1637, "c"=1.8744, "t1"=0.4722,  "t2"=0.3249, "s1"=0.1033, "s2"=0.0371,"m"=0.1072, "s"=0.1934, "f"= 0.8042,  "e"= 0.0174, "w"=0.0113, "i"=0.0332, "v"=0.038)  uly_p<- if(yrs<5) yrs else 5  a_p<- ((1-(1+r)^(-uly_p))/r)  totalCost<- with(ingr, ((p1+s1)/a_p*uly_p+(p2+s2)*(yrs-1)+m*yrs+(t1/a_p*uly_p+  t2*(yrs-1)+(k+p+v+(e+i+s+(c+f+w)*cov)*rnds)*yrs)*target)*pop)  return(totalCost)  }  calc_IRS_Costs(5,360000,0.5,1,0.90,0.03) | | | | |

**Table S8. Average annual financial and economic cost per output by intervention: reference implementation (USD, 2014)**

| Number of years | Financial cost | | | | Economic cost | | | |
| --- | --- | --- | --- | --- | --- | --- | --- | --- |
|  | RR | RACD | MDA | IRS | RR | RACD | MDA | IRS |
| 1 | 6.05 | 33.31 | 2.35 | 3.81 | 8.40 | 39.60 | 2.72 | 4.57 |
| 5 | 4.82 | 20.20 | 2.19 | 3.49 | 6.73 | 23.36 | 2.52 | 4.12 |

Intervention costs per output reflect reference implementation presented in Table 1 above and Additional file 2. The denominator (unit of output) varies by intervention: for RR the estimate represents cost per case reported; for RACD – cost per index case followed-up; for MDA – cost per person treated per round; for IRS – cost per person protected per round. Estimates in the first row show costs incurred in the first year (*i.e.* the year the intervention is first introduced), assuming the intervention is only to be deployed for one year. The second row gives the average annual economic cost assuming each intervention is implemented annually for five years. * Under the reference implementation assumed 50% of the population were targeted by IRS (the denominator refers to the total population). Equivalent cost summaries per capita are reported in Table 2. Costs by implementation stage and cost structure are reported in Additional file 1, Tables S9-S10.

**Table S9. Average annual financial cost and cost structure by intervention: reference implementation (USD, 2014)**

|  | Total costs, $ | | | | Cost profile, % | | | |
| --- | --- | --- | --- | --- | --- | --- | --- | --- |
|  | Rapid Reporting | Reactive Case Detection | Mass Drug Administration | Insecticide Residual Spraying | Rapid Reporting | Reactive Case Detection | Mass Drug Administration | Insecticide Residual Spraying |
| Planning | 1’309 | 1’930 | 1’935 | 5’603 | 2.36 | 0.83 | 0.14 | 0.99 |
| Procurement | 194 | 120’308 | 1’082’706 | 335’998 | 0.35 | 51.70 | 80.82 | 59.48 |
| Distribution | 352 | 3’105 | 12’887 | 602 | 0.63 | 1.33 | 0.96 | 0.11 |
| Storage | 0 | 0 | 0 | 513 | 0.00 | 0.00 | 0.00 | 0.09 |
| Training | 826 | 34’557 | 69’135 | 53’912 | 1.49 | 14.85 | 5.16 | 9.54 |
| Sensitisation | 0 | 5’089 | 14’853 | 15’399 | 0.00 | 2.19 | 1.11 | 2.73 |
| Program management and supervision | 39’133 | 13’208 | 30’689 | 25’167 | 70.44 | 5.68 | 2.29 | 4.46 |
| Implementation | 9’407 | 53’217 | 127’440 | 113’952 | 16.93 | 22.87 | 9.51 | 20.17 |
| Other | 4’334 | 1’300 | 0 | 13’736 | 7.80 | 0.56 | 0.00 | 2.43 |
| Total | 55’556 | 232’713 | 1’339’645 | 564’883 | 100 | 100 | 100 | 100 |

**Table S10. Average annual economic cost and cost structure by intervention: reference implementation (USD, 2014)**

|  | Total costs, $ | | | | Cost profile, % | | | |
| --- | --- | --- | --- | --- | --- | --- | --- | --- |
|  | Rapid Reporting | Reactive Case Detection | Mass Drug Administration | Insecticide Residual Spraying | Rapid Reporting | Reactive Case Detection | Mass Drug Administration | Insecticide Residual Spraying |
| Planning | 2’667 | 4’505 | 7’196 | 12’326 | 3.44 | 1.67 | 0.47 | 1.85 |
| Procurement | 1’061 | 120’510 | 1’082’706 | 337’514 | 1.37 | 44.78 | 70.27 | 50.54 |
| Distribution | 450 | 3’697 | 15’230 | 716 | 0.58 | 1.37 | 0.99 | 0.11 |
| Storage | 0 | 65 | 1’066 | 11’596 | 0.00 | 0.02 | 0.07 | 1.74 |
| Training | 2’501 | 46’334 | 85’452 | 65’353 | 3.22 | 17.22 | 5.55 | 9.79 |
| Sensitisation | 0 | 6’193 | 16’968 | 18’821 | 0.00 | 2.30 | 1.10 | 2.82 |
| Program management and supervision | 42’283 | 22’454 | 66’334 | 73’417 | 54.52 | 8.34 | 4.31 | 10.99 |
| Implementation | 19’369 | 62’586 | 265’808 | 130’275 | 24.97 | 23.26 | 17.25 | 19.51 |
| Other | 9’226 | 2’768 | 0 | 17’786 | 11.90 | 1.03 | 0.00 | 2.66 |
| Total | 77’557 | 269’113 | 1’540’762 | 667’804 | 100 | 100 | 100 | 100 |

| density | **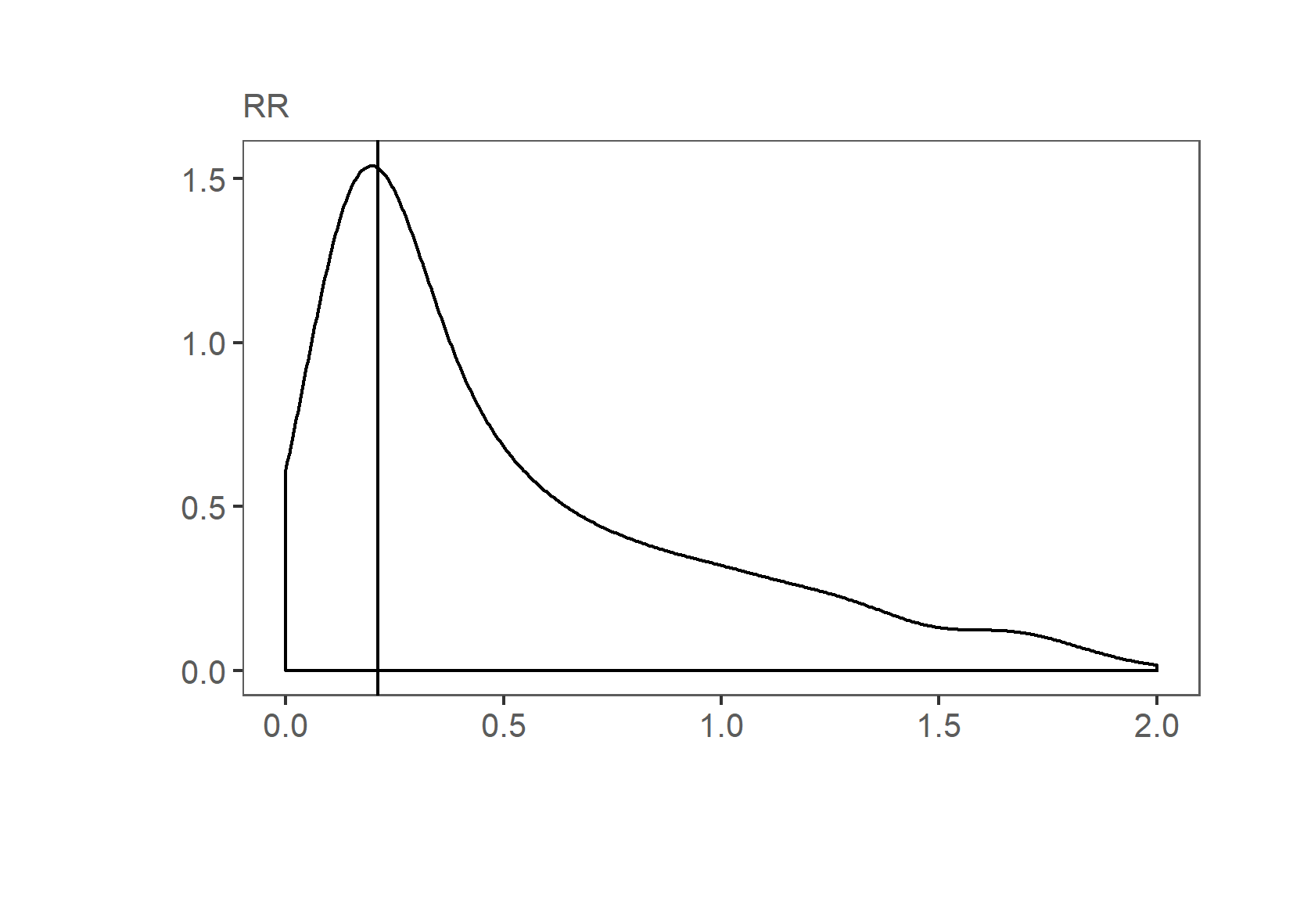** | **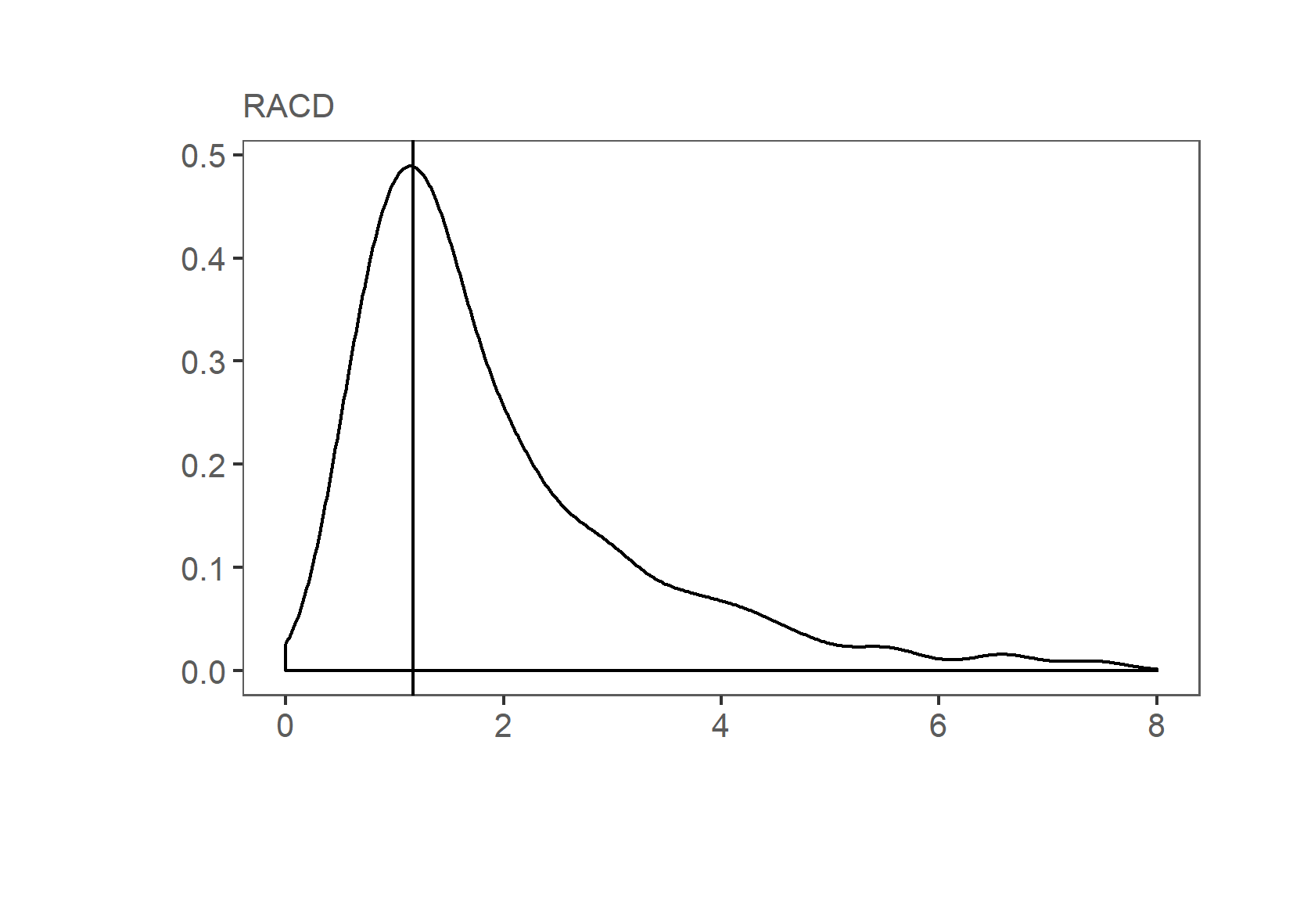** |
| --- | --- | --- |
|  | **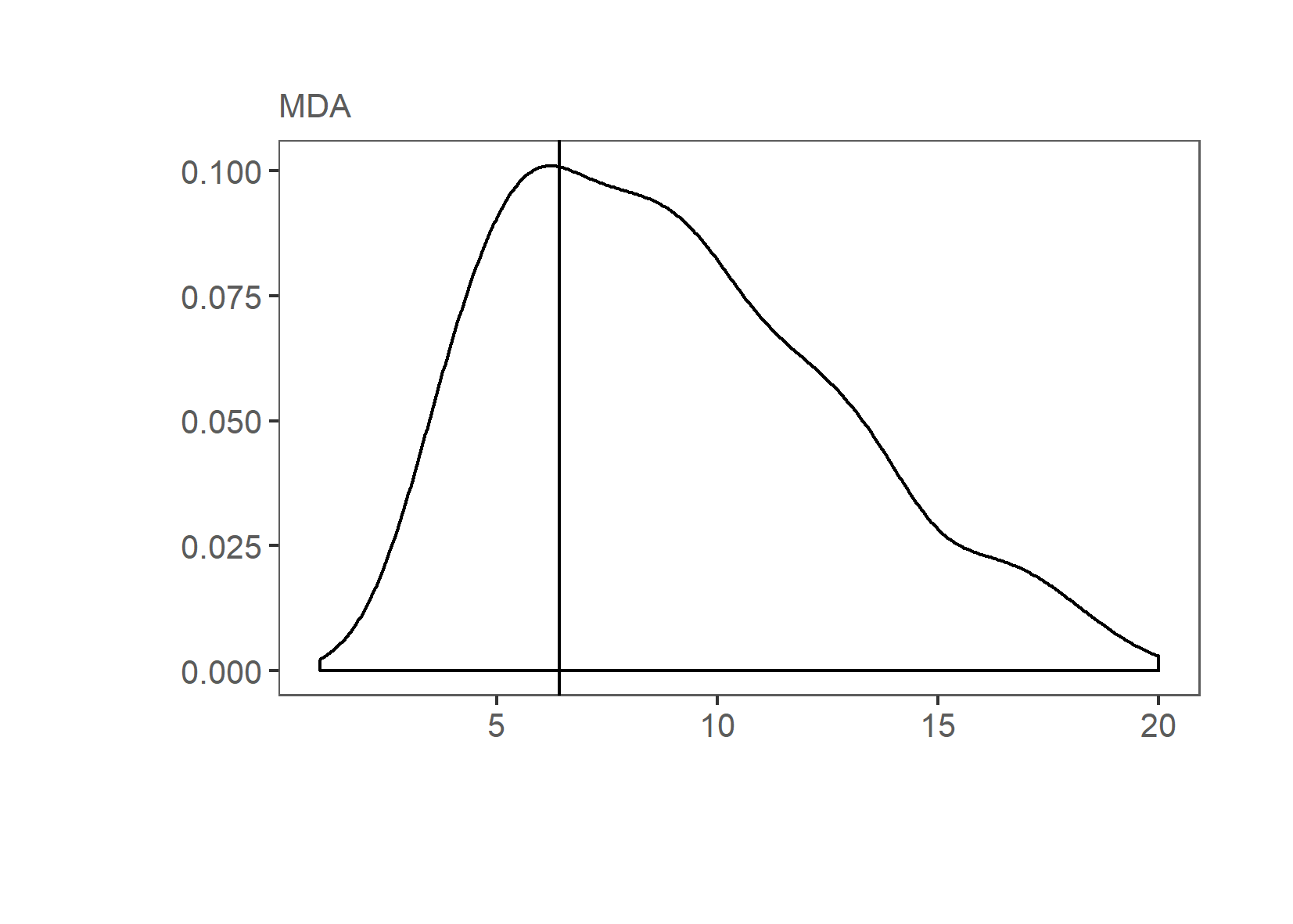** | **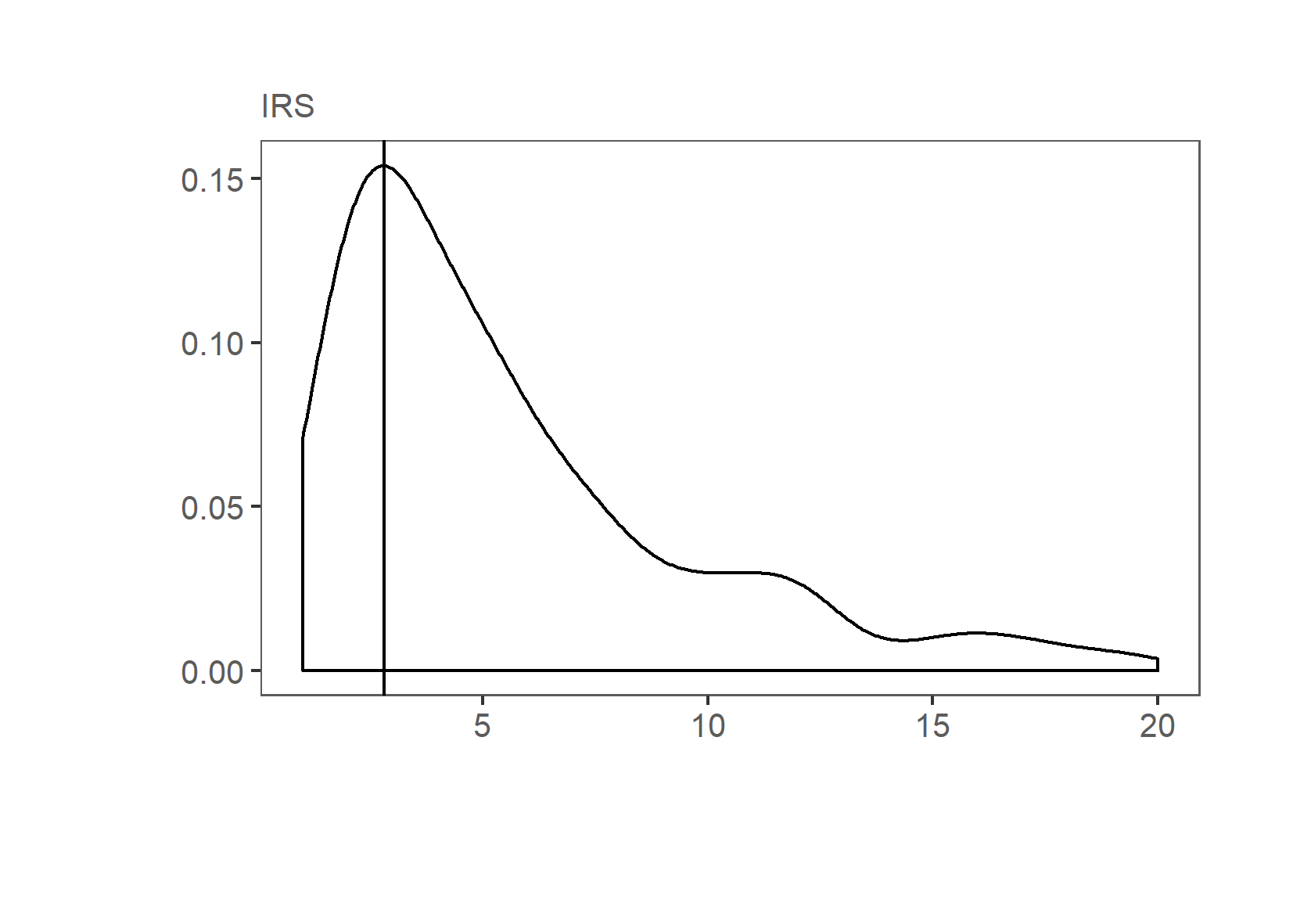** |
|  | cost per capita per year (USD) | |

**Figure S1. Bootstrap analysis: density plots of average annual economic cost per capita (five years) estimated from sampled parameters**

The plots show Kernel distribution of estimated costs per capita obtained from 500 model parameter sets simultaneously sampled 10,000 times from a uniform distribution within the corresponding parameter range (Additional file 3). Lines indicate mode value of the density distribution.

RR= Rapid Reporting; RACD= Reactive Case Detection; MDA= Mass Drug Administration; IRS= Indoor Residual Spraying

| density | **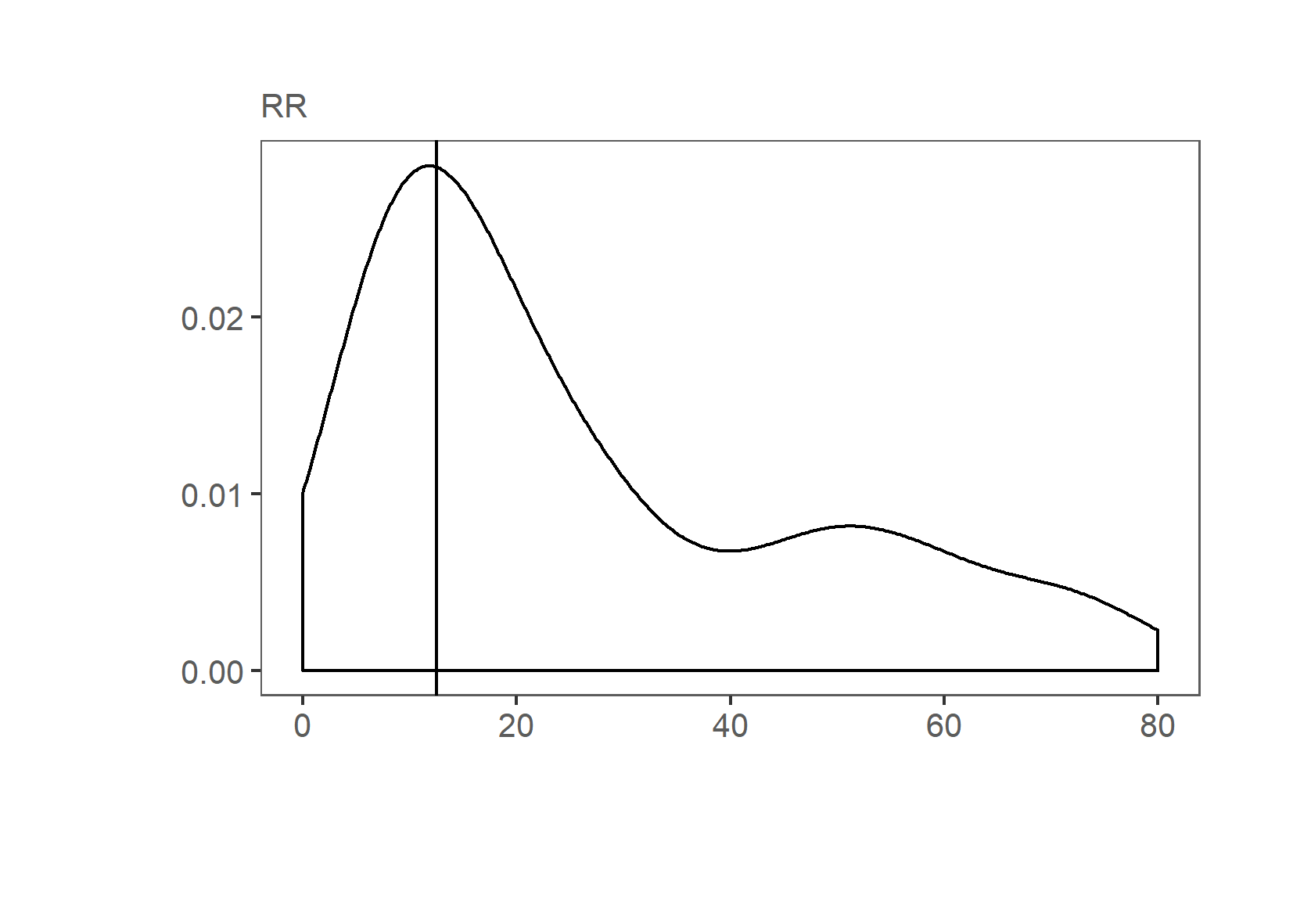** | **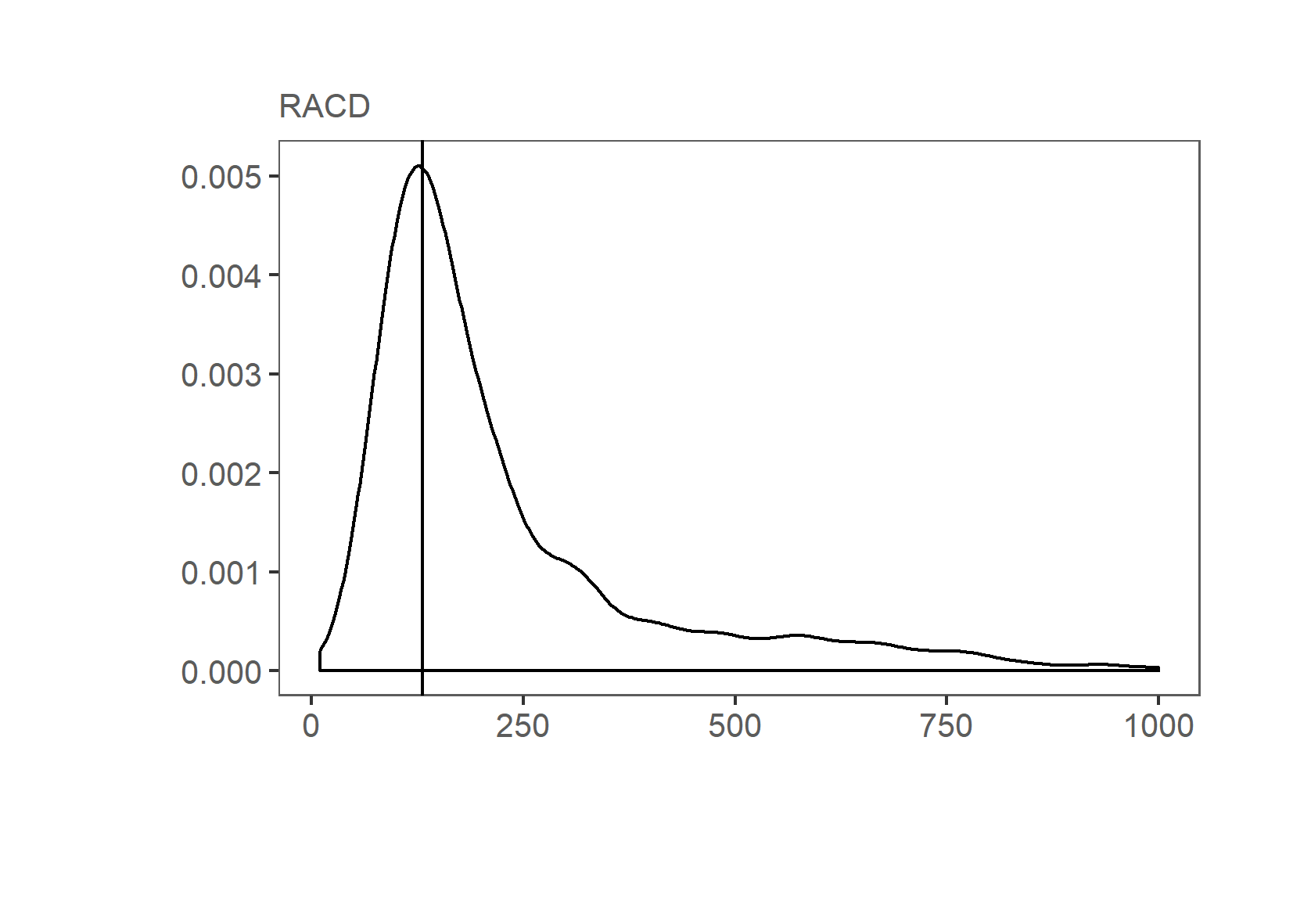** |
| --- | --- | --- |
|  | **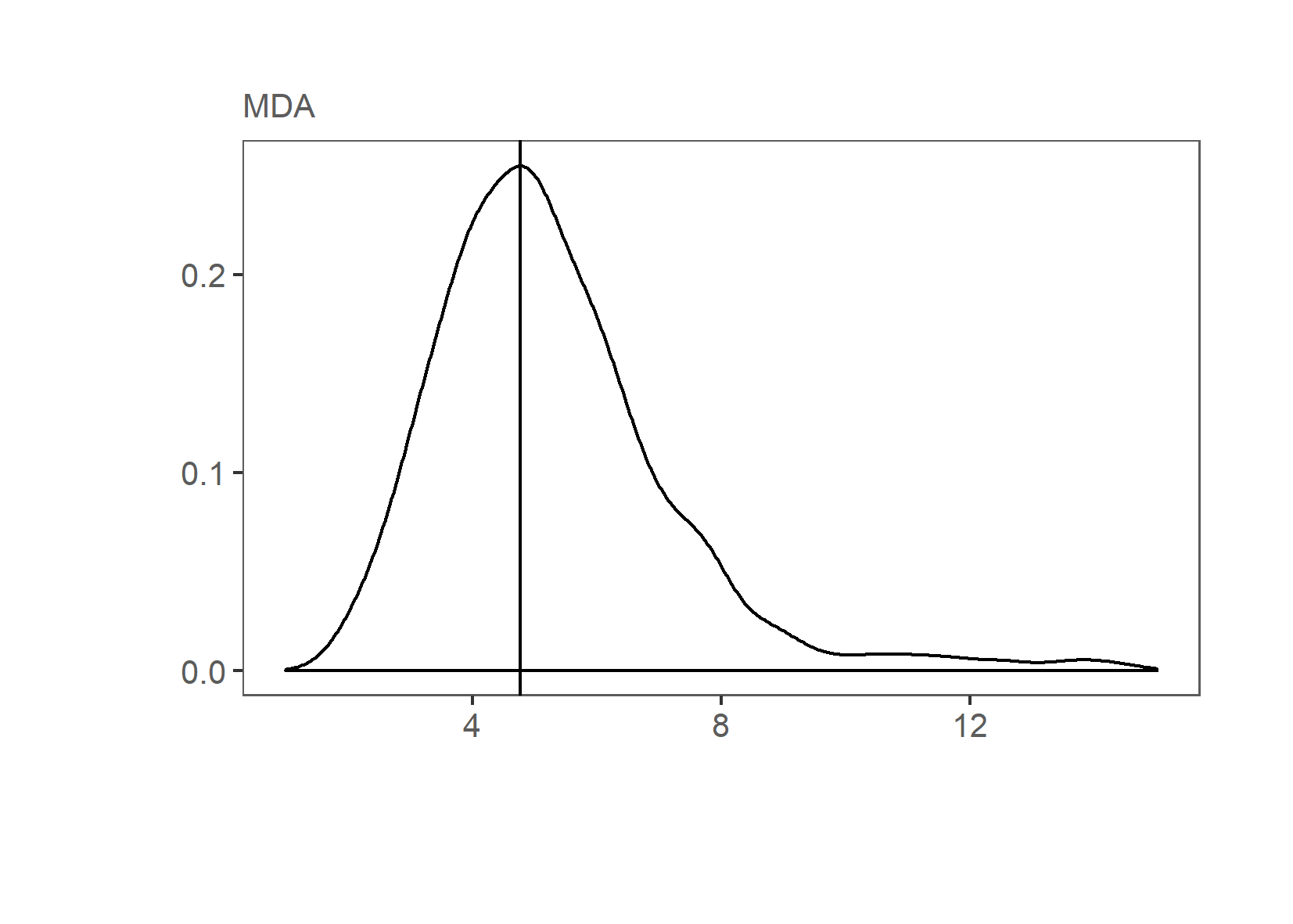** | **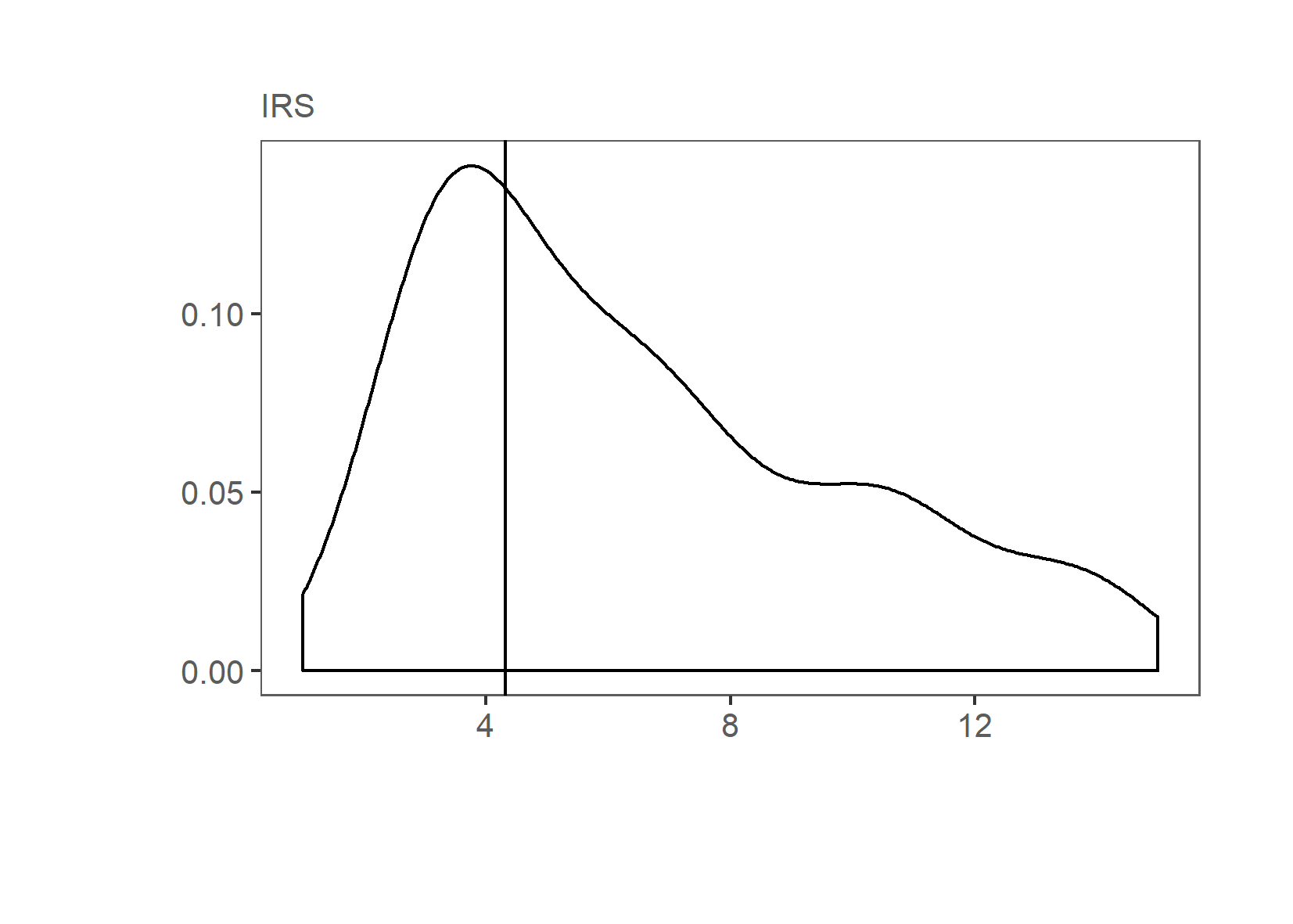** |
|  | cost per capita per year (USD) | |

**Figure S2. Bootstrap analysis: density plots of average annual economic cost per output (five years) estimated from sampled parameters**

The plots show Kernel distribution of estimated costs per capita obtained from 500 model parameter sets simultaneously sampled 10,000 times from a uniform distribution within the corresponding parameter range (Additional file 3). The denominator (unit of output) varies by intervention: for RR the estimate represents cost per case reported; for RACD – cost per index case followed-up; for MDA – cost per person treated per round; for IRS – cost per person protected per round. Lines indicate mode value of the density distribution.

RR= Rapid Reporting; RACD= Reactive Case Detection; MDA= Mass Drug Administration; IRS= Indoor Residual Spraying


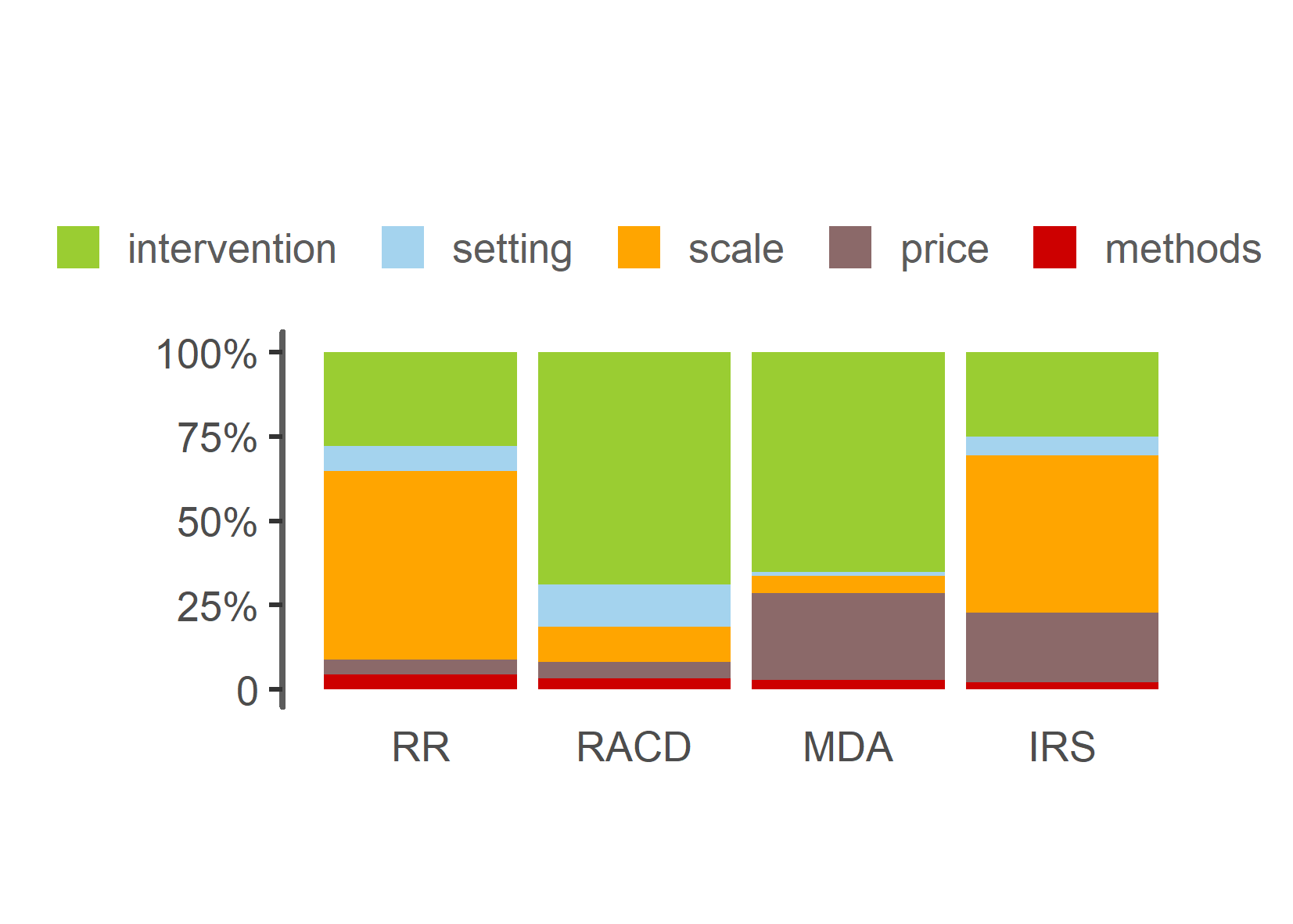


**Figure S3. Bootstrap analysis cost per output: relative contribution by input category**

Color segments of the stacked bars above correspond to the relative joint contribution of model inputs grouped into either of the five categories, describing intervention (green), setting (blue), scale (orange), price level (brown), and methods (red), to intervention unit cost. Model inputs grouped into each category are listed in Additional file 1, Table S2. The proportions represent the joint contribution of model inputs within each category as a fraction of total variation in average annual economic cost per capita explained by the model. These were obtained by regressing cost per output on model inputs sampled from 500 model parameter sets simultaneously drawn 10,000 times from a uniform distribution within the corresponding parameter range (Additional file 3). Model inputs by category are listed in Additional file 1, Table S2. Equivalent distributions for cost per outputs are shown in Figure 2.

RR= Rapid Reporting; RACD= Reactive Case Detection; MDA= Mass Drug Administration; IRS= Indoor Residual Spraying

**Table S11. Values corresponding to reference, minimum and maximum values of parameters highlighted in tornado plots in Figure 3 (highest impact on economic cost per capita when varied singly) by intervention**

| Rapid reporting | | | | Reactive Case Detection | | | | Mass Drug Administration | | | | Insecticide Residual Spraying | | | |
| --- | --- | --- | --- | --- | --- | --- | --- | --- | --- | --- | --- | --- | --- | --- | --- |
| Parameter | Ref | Low | High | Parameter | Ref | Low | High | Parameter | Ref | Low | High | Parameter | Ref | Low | High |
| Reporting days per month | 0.5 | 0.25 | 4.00 | Radius | 5 | 3.6 | 30 | Number of rounds per year | 2 | 1 | 4 | People per structure | 5 | 2 | 11 |
| Allocation RR | 20% | 20% | 100% | Per-diem CHW | $0 | $0 | $15 | Antimalarial drug | $1.64 | $0.82 | $3.27 | Number of rounds | 1 | 1 | 2 |
| Module | $114000 | $57000 | $228000 | Cart, daily rental | $0 | $0 | $12.6 | Persons reached per CHW pair per day | 75 | 20 | 75 | Insecticide, per l | $29.7 | $14.9 | $59.4 |
| Module ULY | 10 | 5 | 20 | Wages CHW, daily | $0.36 | $0 | $11 | MDA coverage | 85% | 50% | 95% | Insecticide per structure | 300 | 200 | 600 |
| Server overheads, yearly | $50000 | $25000 | $100000 | *Pf*PR, all ages | 4% | 1% | 5% | Wages nurse, monthly | $500 | $250 | $1000 | Structures per operator per day | 10 | 5 | 32 |
| Supervision central days | $0 | $0 | $15 | CHW per population | 750 | 500 | 3000 | Per-diem CHW | $0 | $0 | $15 | Per-diem spray operator | $10 | $7.84 | $39 |
| Fuel per l | $1.29 | $0.65 | $2.58 | Proportion followed-up | 100% | 20% | 100% | Per-diem nurse | $20 | $10 | $40 | Training district days | 7 | 3.5 | 14 |
| Wages nurse, monthly | $500 | $250 | $1000 | Top-up | $5 | $0 | $10 | Wages CHW, daily | $0.36 | $0 | $11 | Fuel per l | $1.29 | $0.65 | $2.58 |
| Proportion reporting | 100% | 50% | 100% | Access to care provider | 80% | 30% | 85% | Training district days | 4 | 2 | 8 | Central staff | 3 | 1.5 | 6 |
| Discount rate | 3% | 1% | 10% | Diagnostic test | $1.12 | $0.56 | $2.24 | Transportation allowance district | 20 | 10 | 40 | Number of spray operators per team | 7 | 2 | 7 |

|  | \| **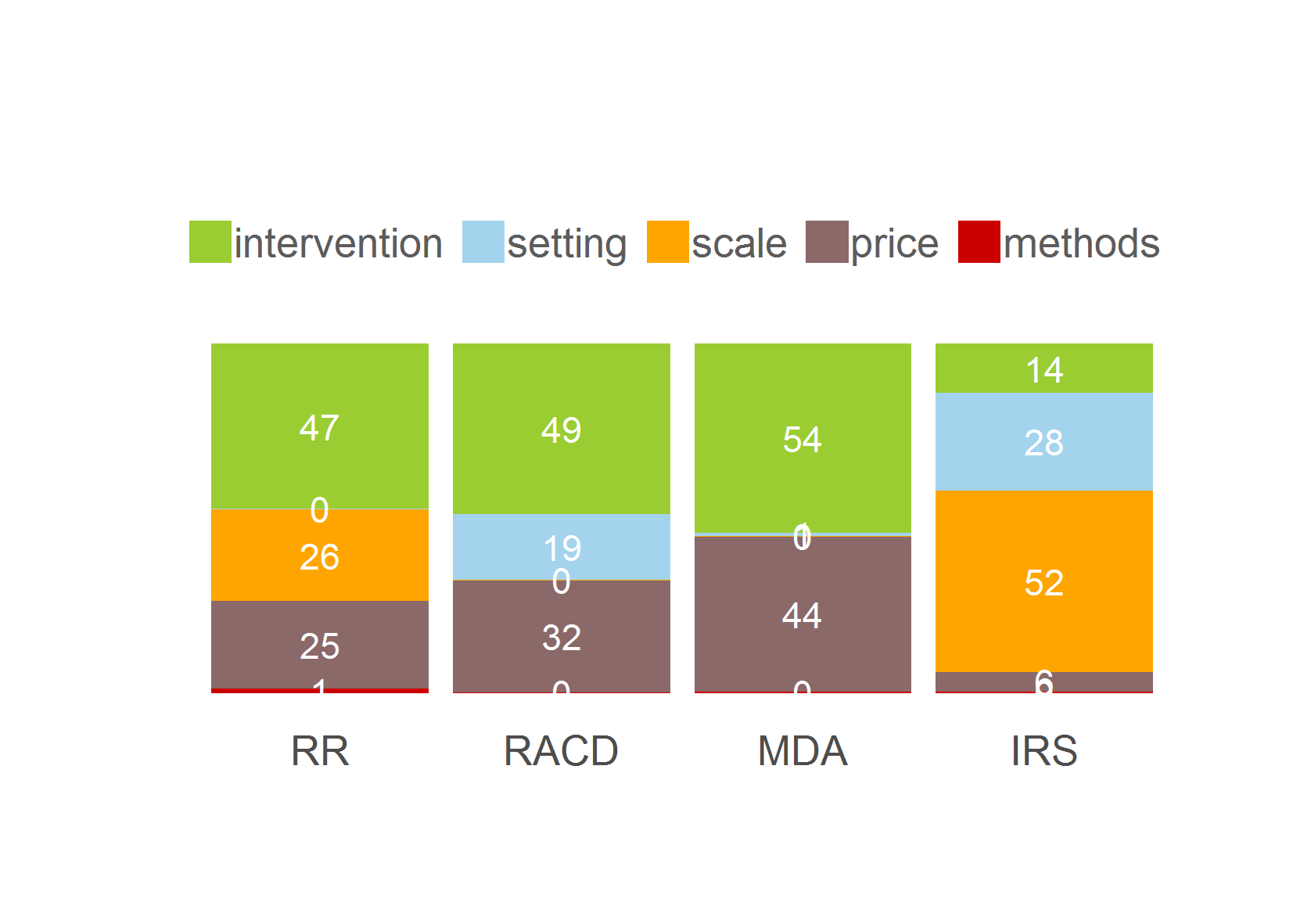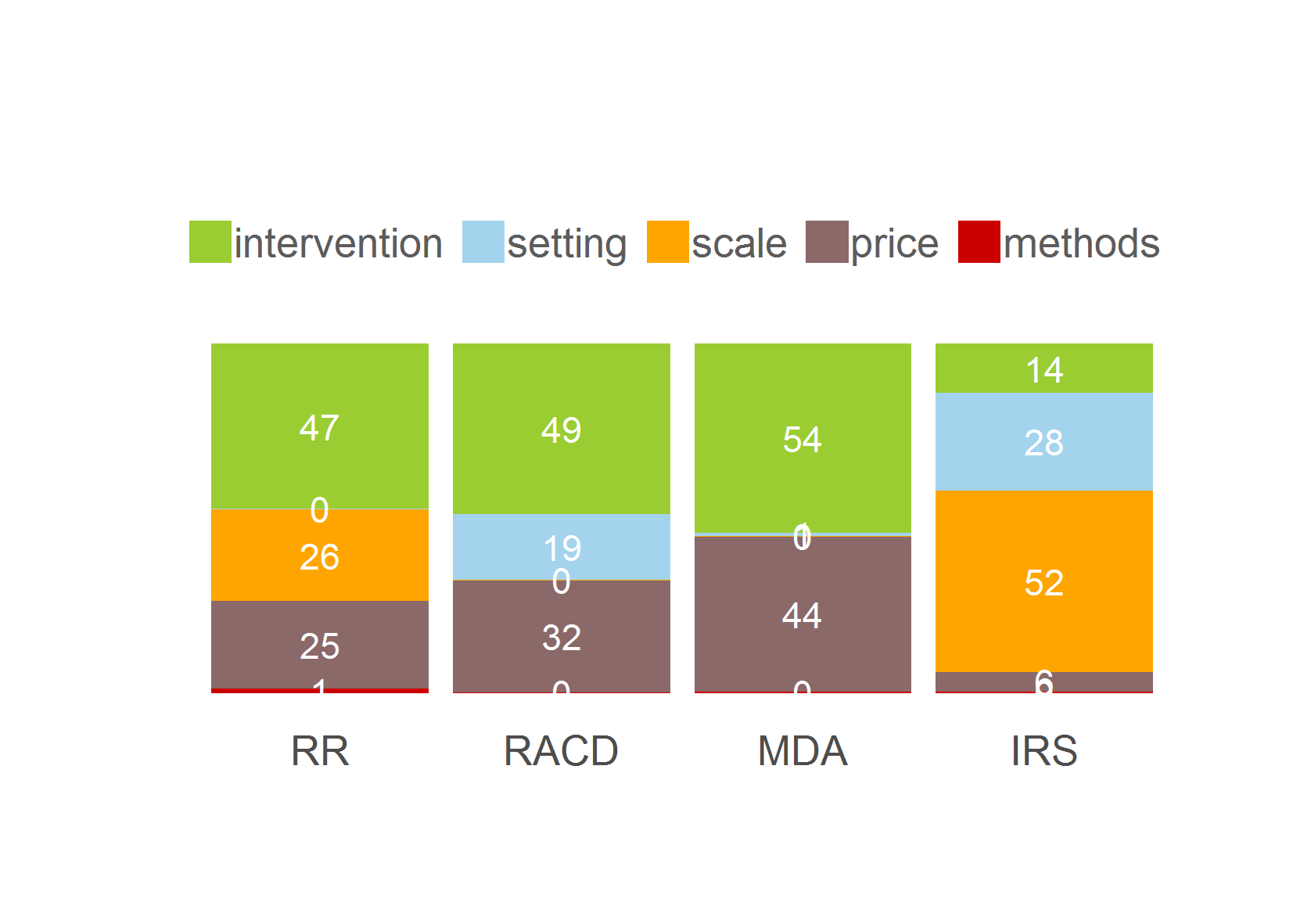** \| \| \| --- \| --- \| \| **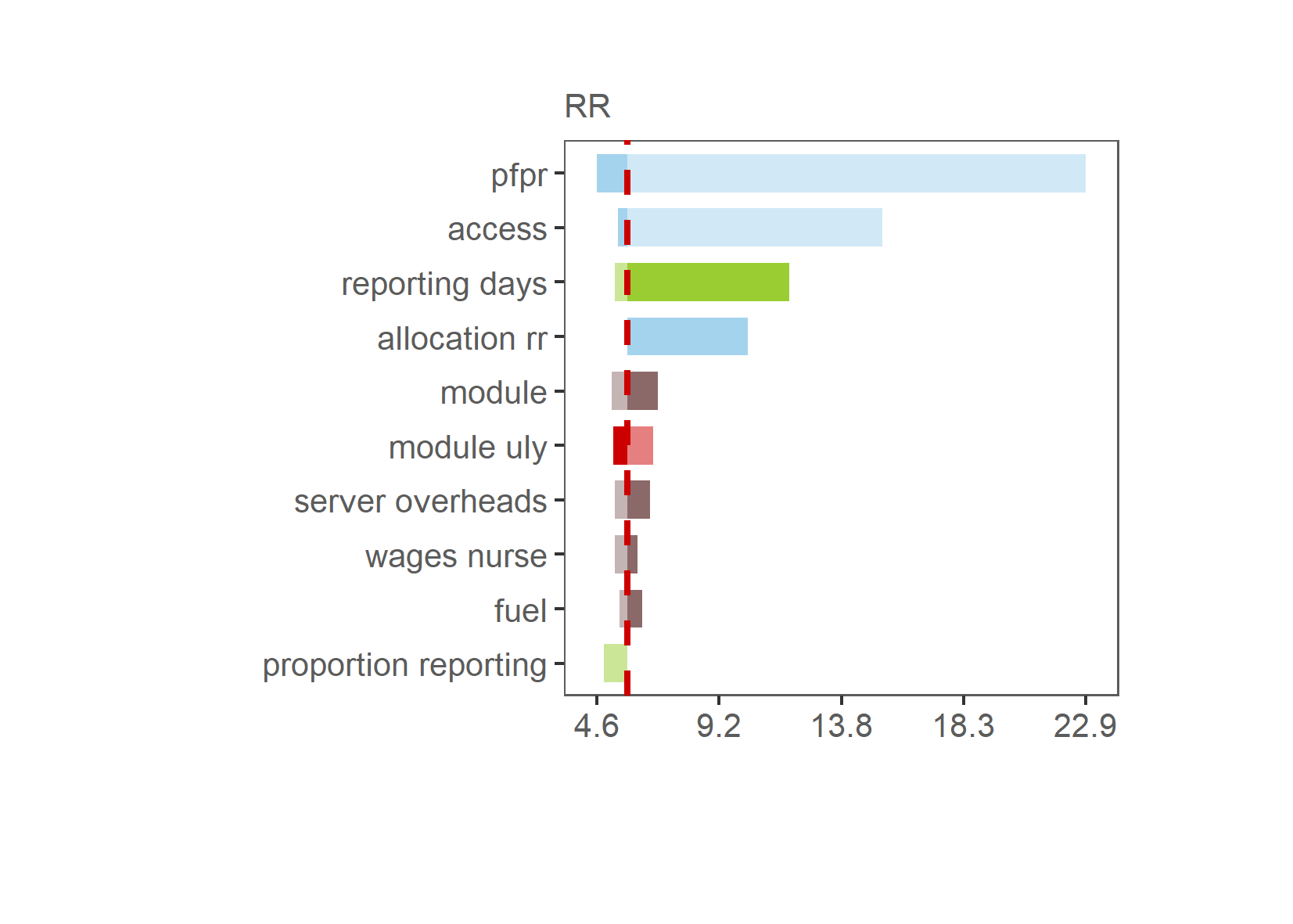** \| **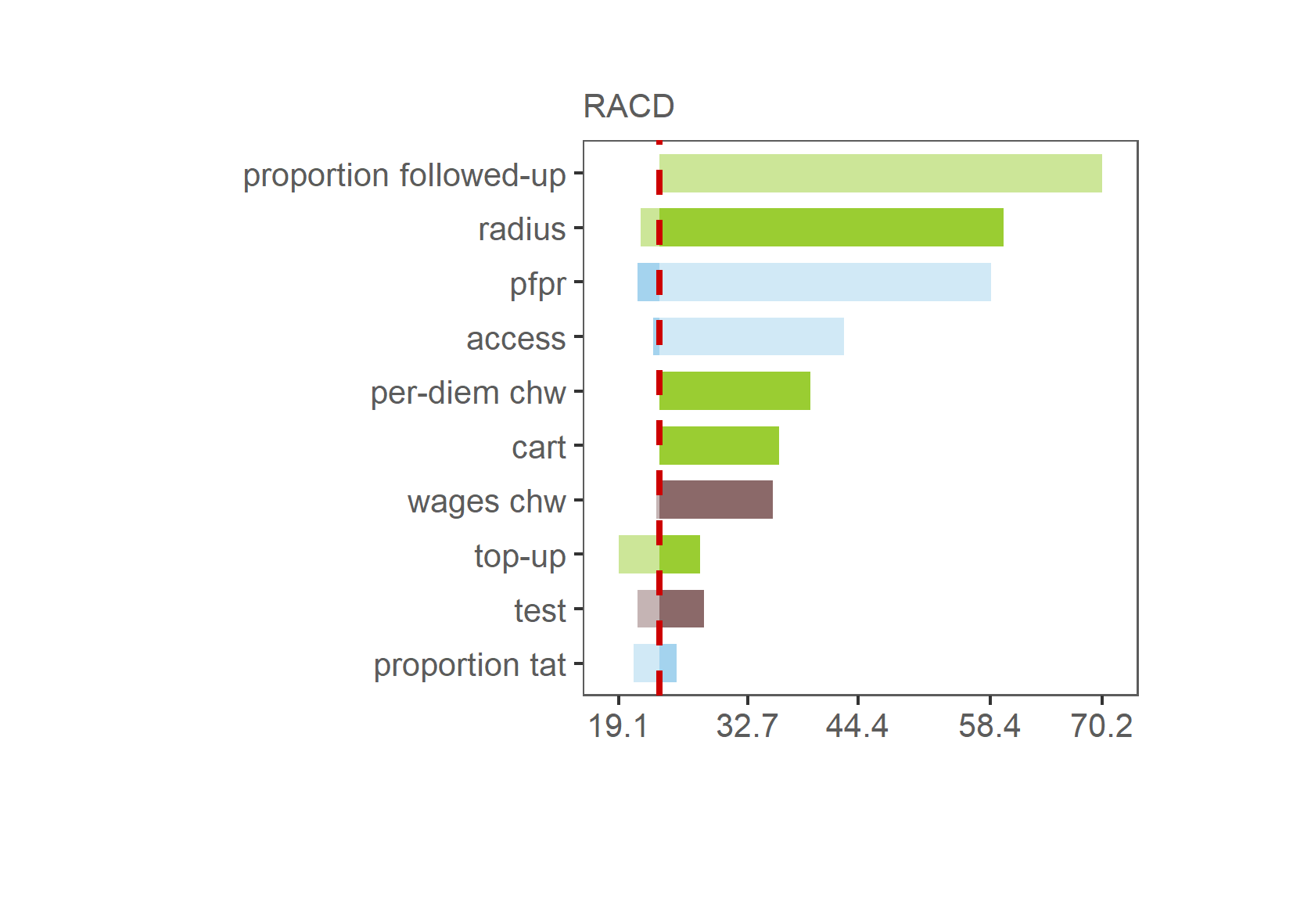** \| \| **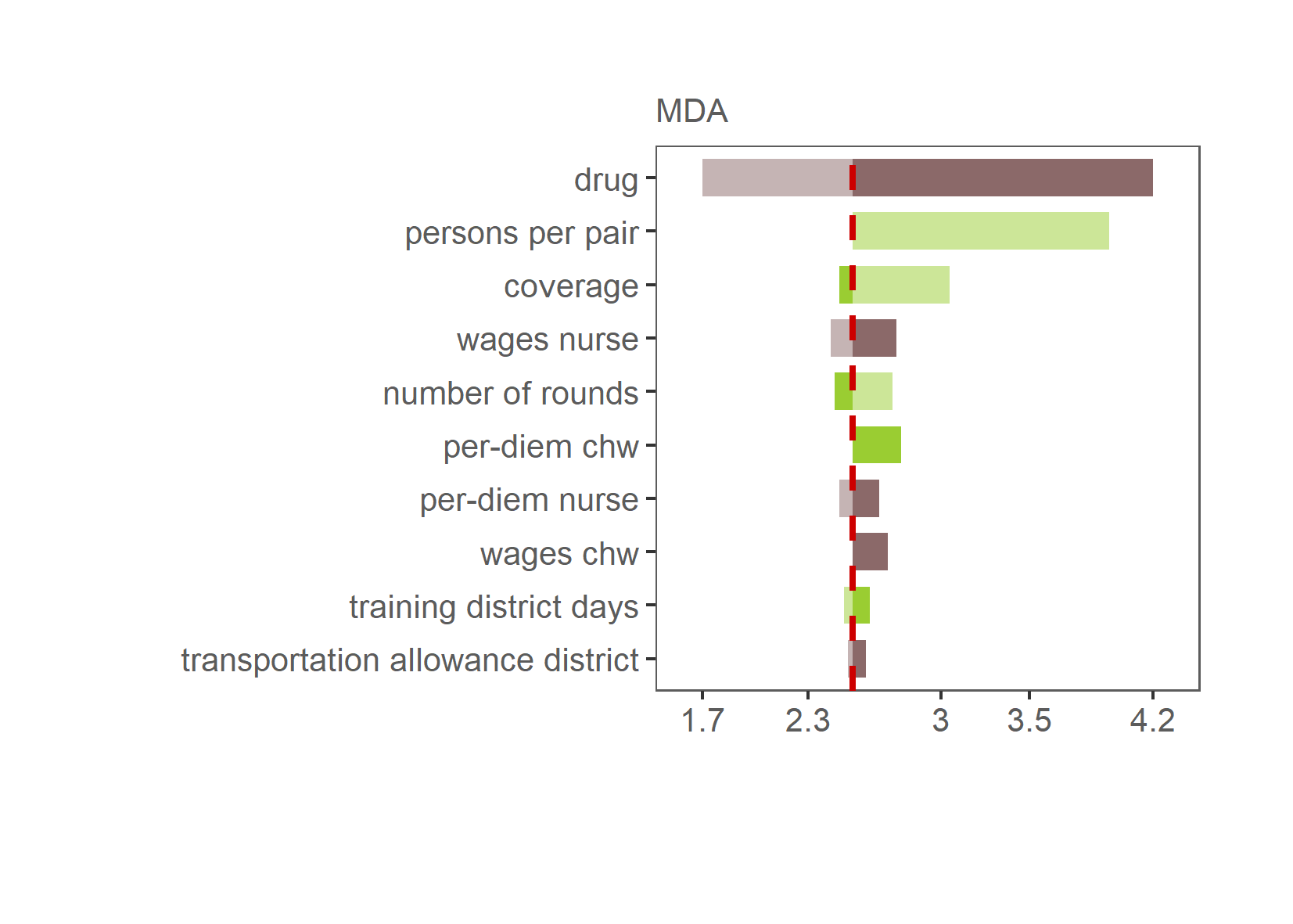** \| **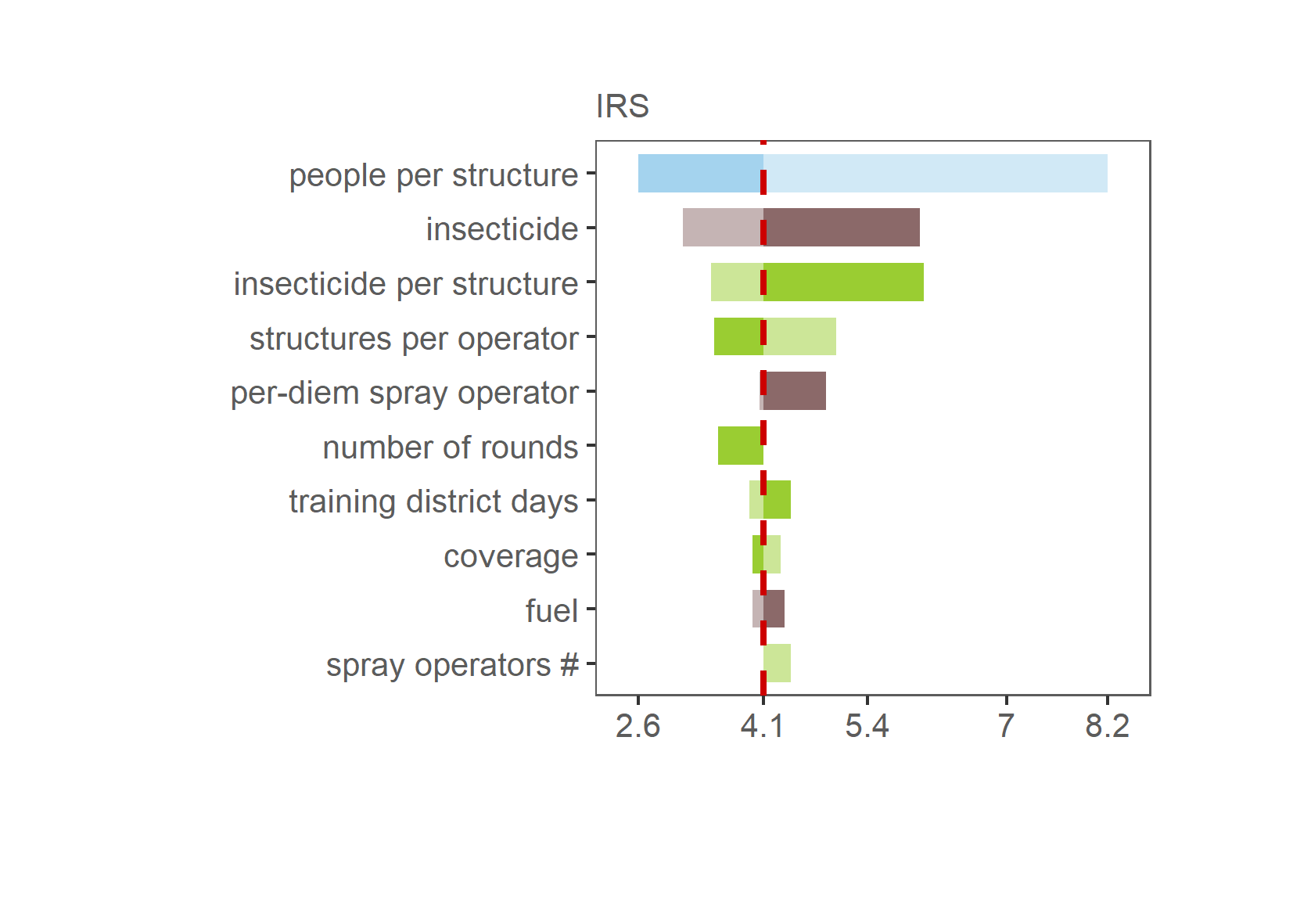** \| \| cost per output (USD) \| \|   **Figure S4. One-way sensitivity analysis of average annual economic cost per output (USD, 2014) at reference implementation***  Tornado plots show top 10 model inputs with the highest impact on intervention unit cost when varied over its’ minimum and maximum while keeping all other inputs at reference values (Additional file 1, Table S11). Bar lengths indicate the value of unit cost at highest – darker shade, and lowest – lighter shade, value of the respective parameter. Bar colour highlights input category. Red dashed lines give the reference estimate. Inputs describing scale of implementation (number of people reached) dominate the unit cost defined in terms of cost per capita; tabulations are thus shown only for parameters related to intervention (green), setting (blue), price (brown), and methods (red). The denominator (unit of output) varies by intervention: for RR the estimate represents cost per case reported; for RACD – cost per index case followed-up; for MDA – cost per person treated per round; for IRS – cost per person protected per round. Impact of scale parameters on estimated unit costs is explored in Figure 4, and Additional file 1, Figure S5. * Reference implementation detailed in Table 1, further details in Additional file 1, Table S3 and Additional file 2.  RR= Rapid Reporting; RACD= Reactive Case Detection; MDA= Mass Drug Administration; IRS= Indoor Residual Spraying.  **Table S12. Model inputs and ranges varied by setting in scenario analyses**   \|  \| parameter varied by setting \| good geographic accessibility setting, high capacity \| poor geographic accessibility setting, high capacity \| good geographic accessibility setting, low capacity \| poor geographic accessibility setting, low capacity \| \| --- \| --- \| --- \| --- \| --- \| --- \| \|  \| Geographic and epidemiological setting \| \| \| \| \| \|  \| Distance between administrative areas \| reference \| referencex2 \| reference \| referencex2 \| \|  \| Population density* \| high \| low \| high \| low \| \|  \| *Pf*PR \| 0.01 \| 0.02 \| 0.02 \| 0.05 \| \|  \| Health systems capacity \| \| \| \| \| \|  \| Number of HF per 10,000 population \| reference \| referencex0.8 \| referencex0.5 \| referencex0.2 \| \|  \| Number of people per CHW \| 750 \| 500 \| 1500 \| 3000 \| \|  \| Health seeking for malaria \| 80% \| 80% \| 60% \| 40% \| \|  \| Days of sensitization \| reference \| referencex0.8 \| referencex0.5 \| referencex0.2 \| \|  \| Days of training \| reference \| reference \| referencex0.5 \| referencex0.5 \| \|  \| Days of supervision \| reference \| referencex0.8 \| referencex0.5 \| referencex0.2 \| \|  \| Number of trainees per training \| 40 \| 40 \| 80 \| 80 \| \|  \| Intervention \| \| \| \| \| \| RR \| DHIS2 \| yes \| yes \| no \| no \| \| % allocation to RR \| 20% \| 20% \| 100% \| 100% \| \| % reporting \| 100% \| 80% \| 60% \| 40% \| \| RACD \| % of index cases followed-up \| 100% \| 80% \| 40% \| 20% \| \| Search radius \| 10 \| 5 \| 10 \| 5 \| \| % of population present for TaT \| 80% \| 50% \| 80% \| 50% \| \| Positivity rate around index case \| 0.1 \| 0.2 \| 0.2 \| 0.5 \| \| MDA \| Number of persons treated per day per pair \| 75 \| 50 \| 75 \| 50 \| \| Number of rounds per year \| 2 \| 2 \| 1 \| 1 \| \| % targeted population treated \| 90% \| 90% \| 50% \| 50% \| \| IRS \| Number of spray operators deployed per district \| 36 \| 36 \| 15 \| 15 \| \| Number of structures sprayed per operator per day \| 20 \| 10 \| 20 \| 10 \| \| Insecticide volume used per m2 \| 300 \| 300 \| 600 \| 600 \| \| % rental vehicles \| 0% \| 0% \| 40% \| 40% \| \| % of targeted structures sprayed \| 90% \| 70% \| 70% \| 50% \|   Parameter values for each setting are *assumed* to illustrate the potential magnitude of these correlations and their impact on intervention costs. We did not explicitly model the relationship between population density and service outputs of interventions, rather assumed that lower output will be observed in more remote settings (i.e. lower bound number of persons treated per day, lower bound number of structures sprayed per day were assumed for poor accessibility settings).  IRS= Indoor Residual Spraying; MDA= Mass Drug Administration; RACD= Reactive Case Detection; RR= Rapid Reporting | |
| --- | --- | --- | --- | --- | --- | --- | --- | --- | --- | --- | --- | --- | --- | --- | --- | --- | --- | --- | --- | --- | --- | --- | --- | --- | --- | --- | --- | --- | --- | --- | --- | --- | --- | --- | --- | --- | --- | --- | --- | --- | --- | --- | --- | --- | --- | --- | --- | --- | --- | --- | --- | --- | --- | --- | --- | --- | --- | --- | --- | --- | --- | --- | --- | --- | --- | --- | --- | --- | --- | --- | --- | --- | --- | --- | --- | --- | --- | --- | --- | --- | --- | --- | --- | --- | --- | --- | --- | --- | --- | --- | --- | --- | --- | --- | --- | --- | --- | --- | --- | --- | --- | --- | --- | --- | --- | --- | --- | --- | --- | --- | --- | --- | --- | --- | --- | --- | --- | --- | --- | --- | --- | --- | --- | --- | --- | --- | --- | --- | --- | --- | --- | --- | --- | --- | --- | --- | --- | --- | --- | --- | --- | --- | --- | --- | --- | --- | --- | --- | --- | --- | --- | --- | --- | --- | --- | --- | --- | --- | --- | --- | --- | --- | --- | --- | --- | --- | --- | --- | --- | --- | --- | --- | --- |
| cost per capita, usd | **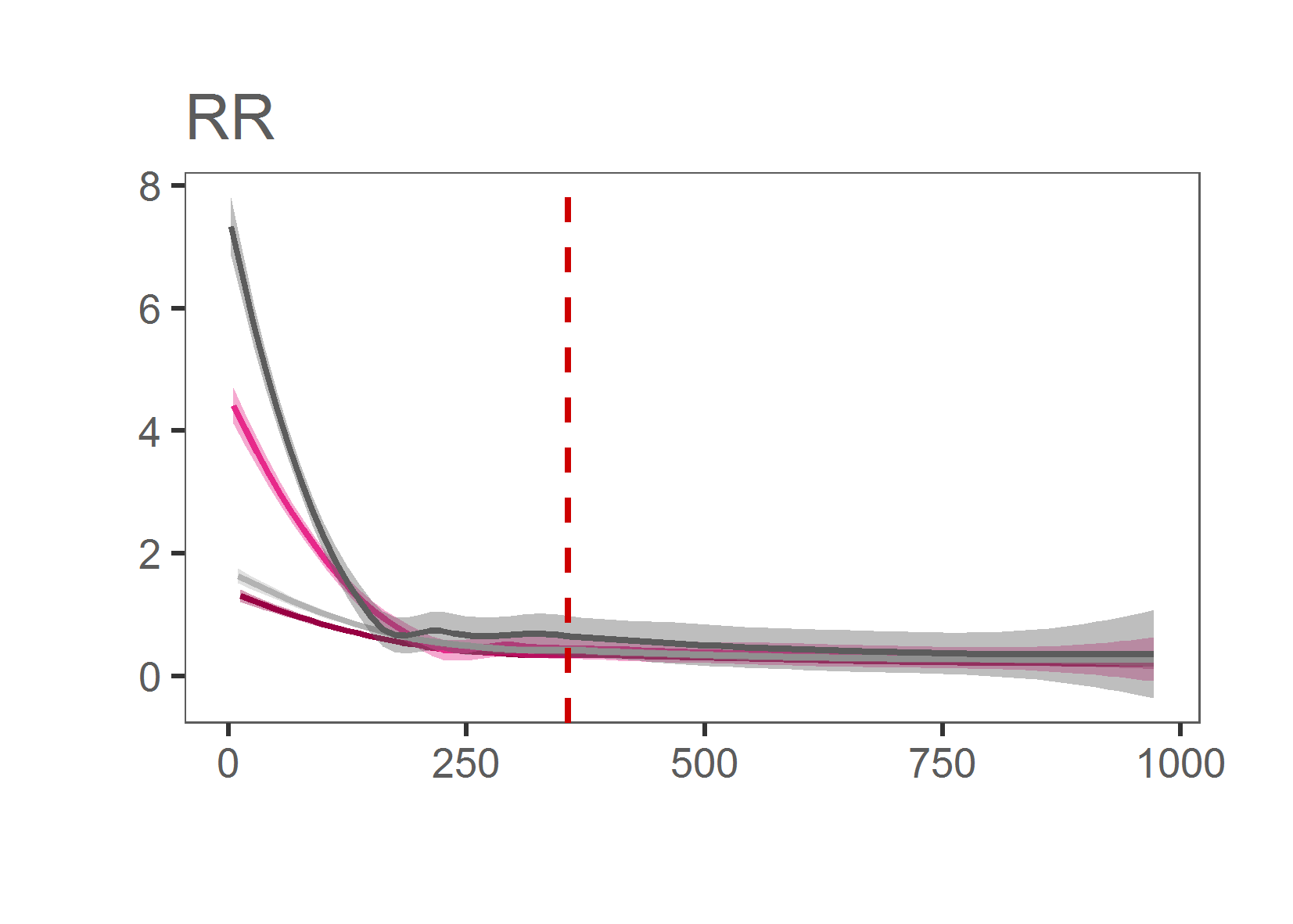** | **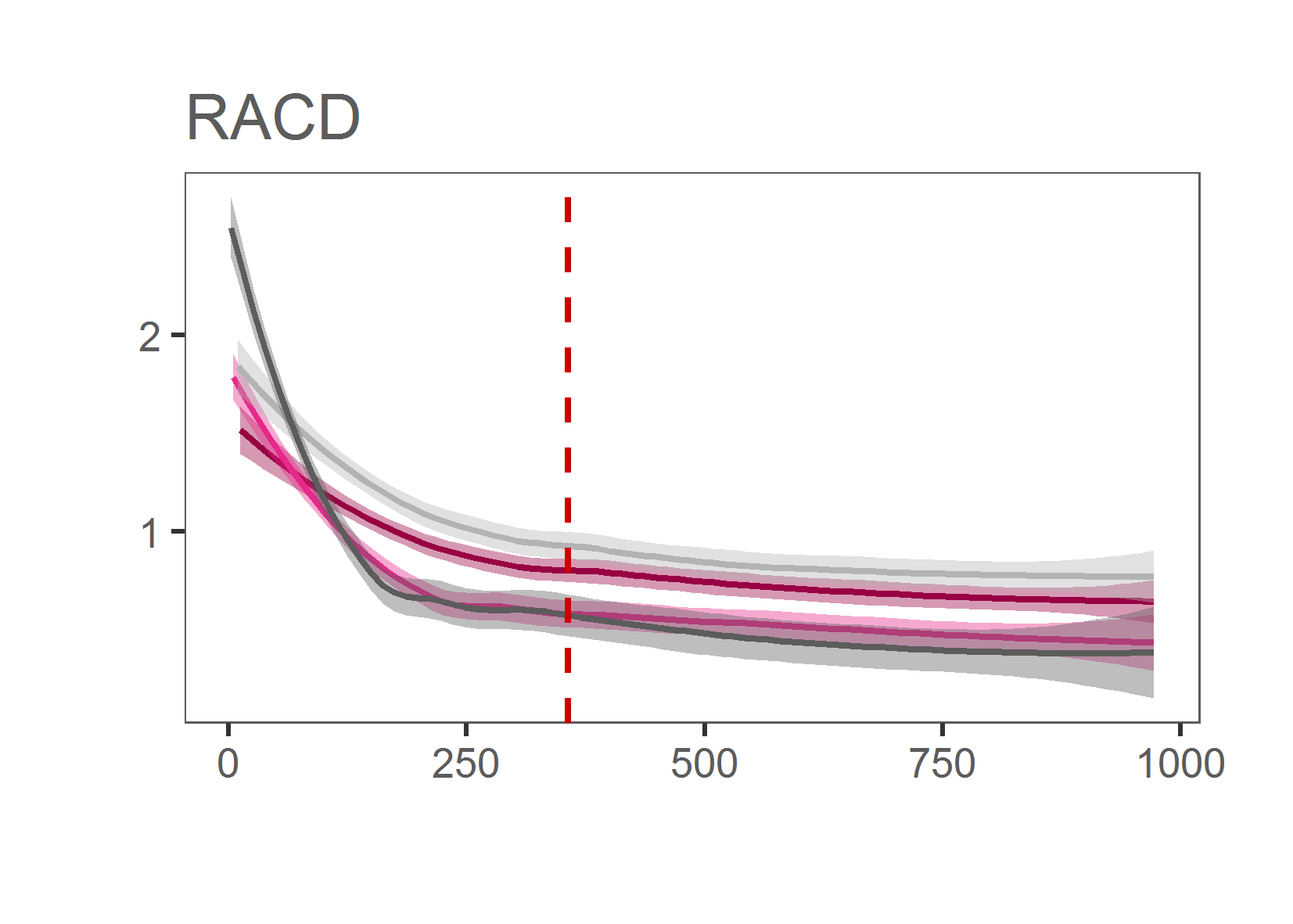** |
|  | **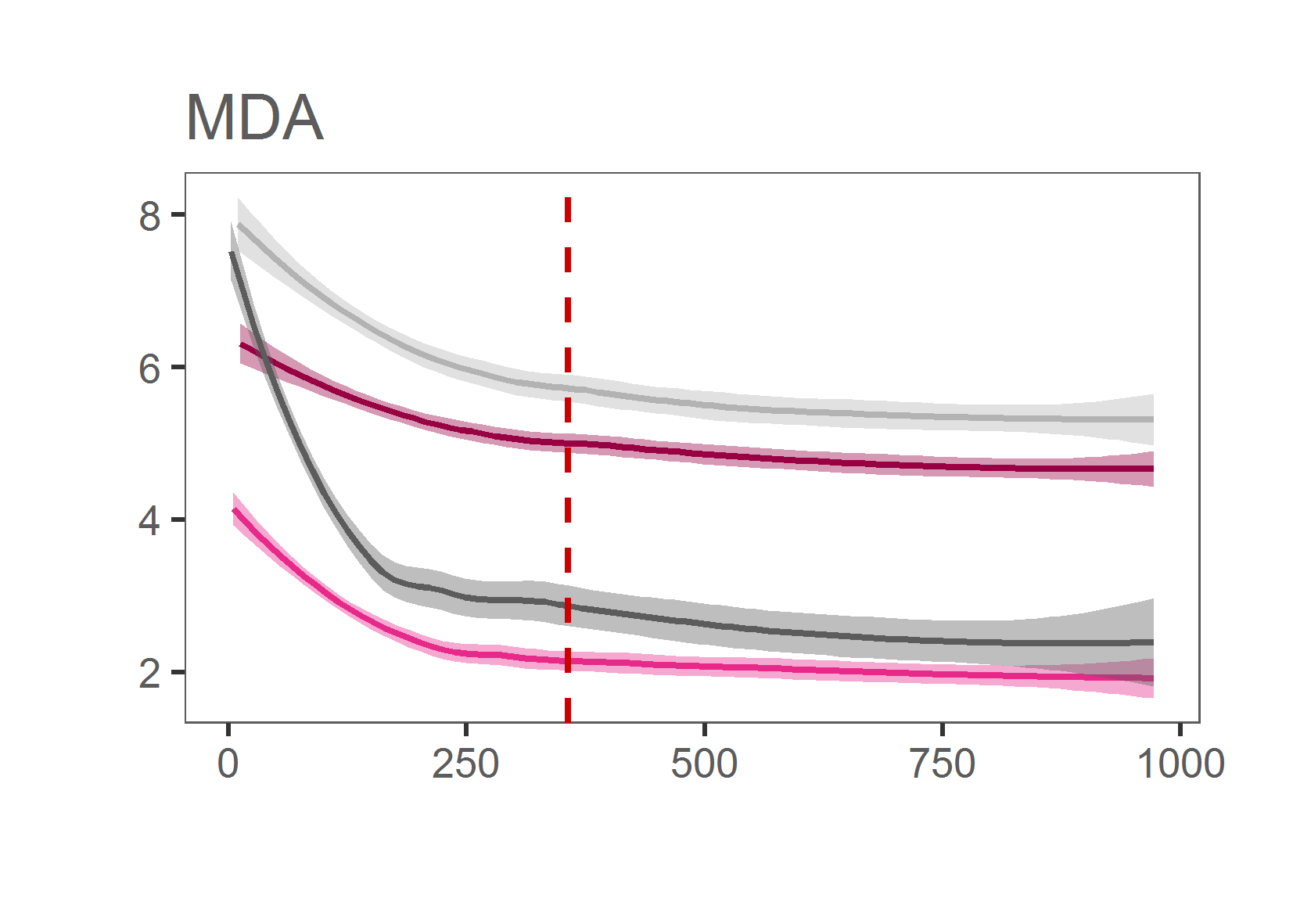** | **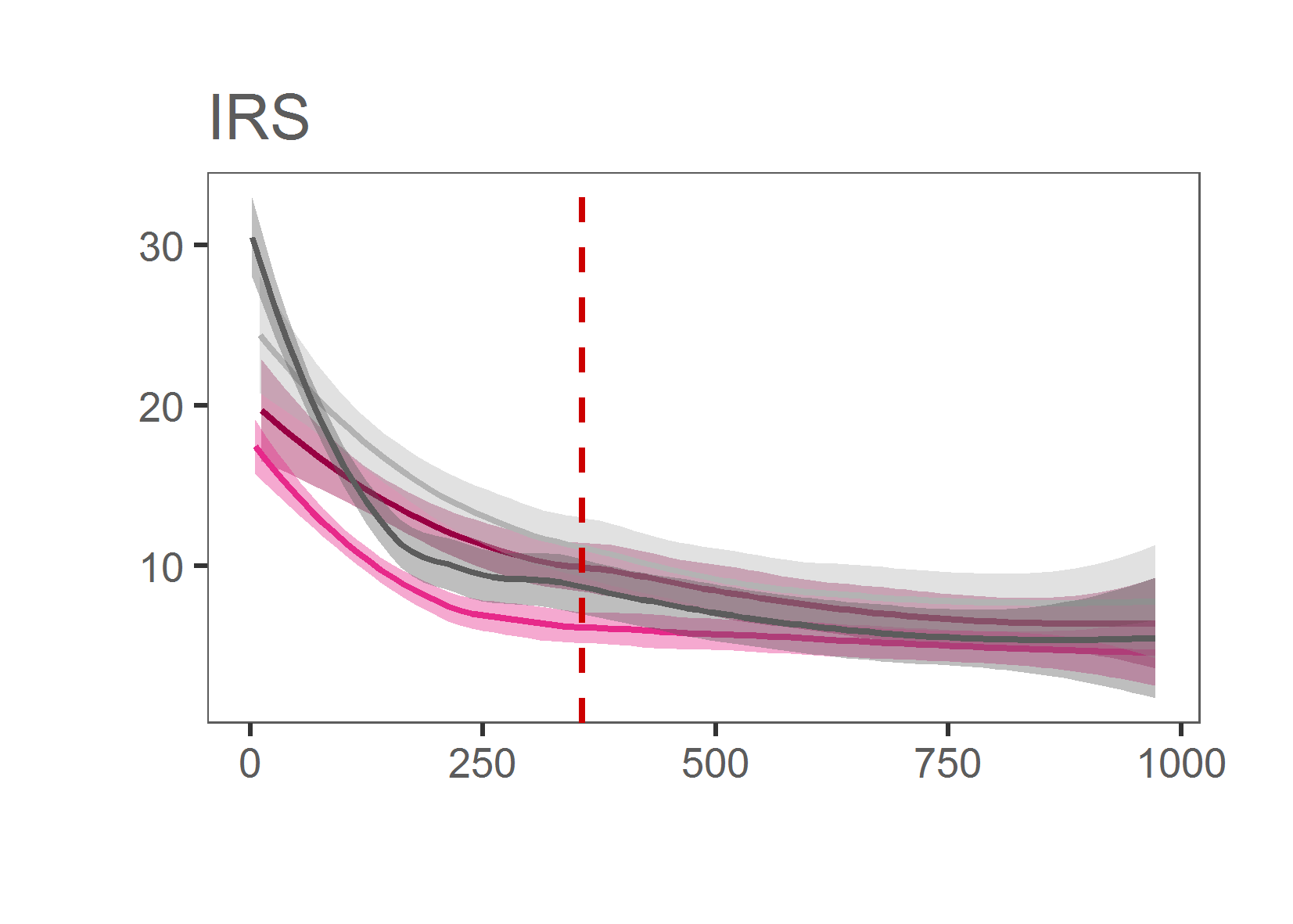** |
|  | population, thousands | |

**Figure S5. Scenario analysis cost per capita per year by setting and scale**

Each curve represents the intervention cost trajectory for the four settings, described in Additional file 1, Table S12, obtained by fitting a Loess curve to cost estimates modelled at various implementation scales. Shaded areas around the curves illustrate variation in the cost estimate due to different ways in which an implementation scale can be represented: by increasing the population size of the HFCA, increasing the number of HFCAs, or increasing the number of districts or regions where the intervention is deployed.

RR= Rapid Reporting; RACD= Reactive Case Detection; MDA= Mass Drug Administration; IRS= Indoor Residual Spraying.

**Table S13. MACEPA reported unit costs from implementation pilots, per capita per year (USD, 2014)**

|  | Rapid Reporting | Case Investigation | Mass Drug Administration* | In-door Residual Spraying |
| --- | --- | --- | --- | --- |
| Zambia | 0.137 | 1.177 | 11.150 | 1.662 |
| Senegal | 0.217 | 0.801 |  |  |
| Ethiopia | 0.388 | 1.632 |  |  |

*The duration of MDA campaign in the pilot averaged 32 days compared to 10 days in the reference implementation; moreover, the scale of the pilots covered 10 districts compared to 3 in the reference implementation, and included a 10 USD per-diem for CHWs distributing drugs that was not costed in the reference implementation.

Source: MACEPA/PATH “Evaluating the costs of implementing interventions and surveillance systems designed to achieve and maintain malaria elimination. Final report.” (2015)

**Table S14. Average annual economic cost of running MDA per capita in Zambia pilots: MACEPA reported vs. standardized (USD, 2014)**

|  | MACEPA report | | Model mapped to MACEPA scope | | Model full scope | |
| --- | --- | --- | --- | --- | --- | --- |
|  | Total | % | Total | % | Total | % |
| Planning | - | - | - | - | 0.984 | 7% |
| Procurement | 2.097 | 19% | 2.414 | 27% | 2.760 | 19% |
| Storage and distribution | 0.021 | 0% | 0.028 | 0% | 0.048 | 0% |
| Training | 3.339 | 30% | 3.195 | 36% | 3.973 | 27% |
| Community sensitization | 0.315 | 3% | 0.312 | 4% | 0.553 | 4% |
| Program management and supervision | 4.180 | 37% | 1.539 | 17% | 4.456 | 30% |
| Implementation (cMDA) | 1.287 | 12% | 1.385 | 16% | 2.135 | 14% |
| Total | 11.150 |  | 8.873 |  | 14.908 |  |

Source: MACEPA/PATH “Evaluating the costs of implementing interventions and surveillance systems designed to achieve and maintain malaria elimination. Final report.” (2015)

While standardized unit costs are relatively similar in absolute terms to unit costs in MACEPA report, these represent a very different scope of resources captured with the models presented here. To appreciate this, first compare cost estimates under “MACEPA report” to “Swiss TPH models MACEPA mapped scope”. The first column presents cost estimates as these appear in MACEPA report, the second set of estimates were produced with Swiss TPH costing models mapped into the scope of resources covered in the original analysis of the trial. When moving from first to second set of estimates we fixed any inconsistencies in valuation of resources (i.e. in application of per-diems, transportation, units of measurement (for further details refer to assumptions database)), replaced MACEPA wages and per-diem with the corresponding program wages, and updated vehicle rental costs with NMCP vehicles operational costs. Our costing model does very well replicating unit costs in the report. As expected, the biggest difference is in the two cost categories that are dominated by MACEPA wages and rental vehicles (“Training” and “Program management and supervision”); MACEPA costs accounted for over 20% of the total intervention costs. By design, costing analyses within the MACEPA trials excluded any costs occurring above district level and ignored economic value of health systems infrastructure other than labour. Cost estimates produced with the Swiss TPH methodology address these limitations by explicitly defining and evaluating resource use at higher programmatic levels and valuing the full scope of resources (for economic costs) used to deliver the interventions. Comparing the second set of estimates to the third (i.e. “Swiss TPH models full scope”) shows that the original scope of the costing analysis only captured about 60% of the overall cost of the intervention.

Differences in costs by category are due to the following:

1. “Planning” was not previously evaluated, important for MDA (relatively high contribution to overall costs) as it is an activity that is repeated each year the intervention is deployed
2. Higher cost estimate for “Procurement” reflects wastage on drugs and diagnostics not previously accounted for
3. Higher cost estimate for “Storage and distribution” is due to inclusion of storage and distribution costs at district level, only central level was costed previously
4. “Training” now includes training of supervisors
5. “Supervision” now includes supervision by regional, district, and health facility staff, only MACEPA supervision was costed previously
6. Higher costs under “Implementation” include transportation and lodging allowance for hard to reach areas (as per protocol) during campaign rounds, printing of forms

**Table S15. Costs of RR, RACD, MDA, and IRS from the literature (USD)**

|  | Ref | Country | Costing year | Unit | Scope | Trial or program | Scale (population) | Median cost estimate [range] | Notes |
| --- | --- | --- | --- | --- | --- | --- | --- | --- | --- |
| IRS | [25] | Multiple | 2009 | ppp | F | Mix | Mix | $3.91 [$1.14-$12.87] |  |
| IRS | [26] | Multiple | 2009 | ppp | E | Mix | Mix | $3.41 [$1.14-$6.23] |  |
| IRS | [26] | Multiple | 2018 | ppp | F | Program | Mix | $5.73 [$2.78-$14.23] | Including PMI support |
| IRS | [26] | Zambia | 2018 | ppp | F | Program | 276,343* | $2.78 | Including PMI support |
| IRS | [26] | Tanzania | 2018 | ppp | F | Program | 2,840,927* | $3.79 | Including PMI support |
| IRS | [26] | Benin | 2018 | ppp | F | Program | 1,321,758* | $3.72 | Including PMI support |
| RACD | [27] | Indonesia | 2015 | par | E | Trial | 61,209 | $0.42 |  |
| RR~HMIS | [28] | Multiple | 2006 | pc | F | Program | 30,000,000 | $0.16 [$0.53-$2.99] | Model |
| MDA | [29] | Multiple | 2015 | ppt | F | Mix | 10,000-1,000,000 | $0.10-$2.54 | Meta-regression estimate, net of drugs and community volunteers |
| MDA~SMC | [30] | Senegal | 2010 | ppt | F | Trial | 180,000 | $0.32 | In children <10, three annual rounds per year, at an average coverage of 93%; excluding research-participation incentives |
| MDA~SMC | [30] | Senegal | 2010 | ppt | E | Trial | 180,000 | $0.40 | In children <10, 3 rounds per year, at an average coverage of 93%, door-to-door; excluding research-participation incentives |
| MDA~SMC | [31] | 6 countries, SSA | 2015 | ppt | F | Program | 100,000-1,000,000 | $1.1 [$0.86-$1.52] | In children 3-59 months, 4 rounds per year, coverage of all 4 rounds varies by country from 23% to 70%, door-to-door |

RR= Rapid Reporting; RACD= Reactive Case Detection; MDA= Mass Drug Administration; IRS= Indoor Residual Spraying; HMIS= Health Management Information System; SMC= Seasonal Malaria Chemoprophylaxis; ppp= per person protected; par= per person at risk; pc= per capita; ppt= per person treated; F= financial costs; E= economic costs; * number of people protected

The methodology detailed here produced intervention cost estimates that are consistent with the literature. Our reference estimate for IRS matches the median from a systematic review of economic studies of malaria interventions [25]. Across these, somewhat dated evaluations, cost per person protected averaged 3.91 and 3.41 USD for financial and economic cost with a range between 1.11 and 12.87 USD. More recent analyses of country IRS programs reported cost per person protected at about 5.73 USD [26]. Our estimate is at the lower end of these country evaluations - comparable to costs from Zambia, Tanzania and Benin at about 3 to 4 USD per person protected. To the best of our knowledge, only one other study [27] outside of MACEPA pilots (Additional file 1, Tables S13 and S14, and [7]) evaluated costs of malaria RACD. [27] cited 0.42 USD per person at risk for a community implementation using microscopy and a radius around an index case of 500 meters (an average of 42 people) in a pre-elimination setting in Indonesia. It is difficult, however, to meaningfully compare the two estimates given differences in design, scale of evaluation and incidence between the studies. Cost estimates from similar programs offer informative benchmarks for interventions modelled here but not yet routinely deployed by malaria programs. For instance, operationally, rapid malaria reporting is similar to routine reporting for other diseases and health systems monitoring. [28] valued facility-based health information systems in LMICs at around 0.16 USD per capita with a range between 0.53 and 2.99 USD which is comparable to our average annual economic cost of 0.22 USD per capita. MDA, while not yet routinely deployed for malaria, is an established strategy for control of Neglected Tropical Diseases (NTDs). A recent meta-analysis of this literature estimated economic cost of service delivery per person treated net of drugs at an average of about 0.40 USD with a range between 0.02 and 2.90 [29]. The service delivery component of the economic cost for the reference implementation is 0.75 USD per person treated - well within the range of the literature. The higher estimate from the model, in part, reflects differences in valuation of community resources, specifically, the opportunity cost of community volunteers excluded in [29] were accounted for in this study. Evaluations of seasonal malaria chemoprophylaxis (SMC) offer another informative reference. As MDA, drug distribution for SMC relies on CHWs to administer drugs to; comparable to estimates derived here, net of drugs the service delivery components of large-scale SMC programs were estimated between 0.10 and 2.54 USD per child treated per round [30, 31].

**References**

1. Githinji S, Oyando R, Malinga J, Ejersa W, Soti D, Rono J, Snow RW, Buff AM, Noor AM: **Completeness of malaria indicator data reporting via the District Health Information Software 2 in Kenya, 2011-2015.** *Malar J* 2017, **16:**344.

2. Kariuki JM, Manders EJ, Richards J, Oluoch T, Kimanga D, Wanyee S, Kwach JO, Santas X: **Automating indicator data reporting from health facility EMR to a national aggregate data system in Kenya: An Interoperability field-test using OpenMRS and DHIS2.** *Online J Public Health Inform* 2016, **8:**e188.

3. Kiberu VM, Matovu JK, Makumbi F, Kyozira C, Mukooyo E, Wanyenze RK: **Strengthening district-based health reporting through the district health management information software system: the Ugandan experience.** *BMC Med Inform Decis Mak* 2014, **14:**40.

4. Quan V, Hulth A, Kok G, Blumberg L: **Timelier notification and action with mobile phones-towards malaria elimination in South Africa.** *Malar J* 2014, **13:**151.

5. WHO: **Malaria Surveillance, Monitoring and Evaluation: A reference manual.** 2018.

6. Larsen DA, Chisha Z, Winters B, Mwanza M, Kamuliwo M, Mbwili C, Hawela M, Hamainza B, Chirwa J, Craig AS, et al: **Malaria surveillance in low-transmission areas of Zambia using reactive case detection.** *Malar J* 2015, **14:**465.

7. Larson BA, Ngoma T, Silumbe K, Rutagwera MR, Hamainza B, Winters AM, Miller JM, Scott CA: **A framework for evaluating the costs of malaria elimination interventions: an application to reactive case detection in Southern Province of Zambia, 2014.** *Malar J* 2016, **15:**408.

8. Littrell M, Sow GD, Ngom A, Ba M, Mboup BM, Dieye Y, Mutombo B, Earle D, Steketee RW: **Case investigation and reactive case detection for malaria elimination in northern Senegal.** *Malar J* 2013, **12:**331.

9. Sturrock HJ, Novotny JM, Kunene S, Dlamini S, Zulu Z, Cohen JM, Hsiang MS, Greenhouse B, Gosling RD: **Reactive case detection for malaria elimination: real-life experience from an ongoing program in Swaziland.** *PLoS One* 2013, **8:**e63830.

10. Searle KM, Hamapumbu H, Lubinda J, Shields TM, Pinchoff J, Kobayashi T, Stevenson JC, Bridges DJ, Larsen DA, Thuma PE, et al: **Evaluation of the operational challenges in implementing reactive screen-and-treat and implications of reactive case detection strategies for malaria elimination in a region of low transmission in southern Zambia.** *Malar J* 2016, **15:**412.

11. WHO: **A framework for malaria elimination.** 2017.

12. Hodges MH, Sonnie M, Turay H, Conteh A, Maccarthy F, Sesay S: **Maintaining effective mass drug administration for lymphatic filariasis through in-process monitoring in Sierra Leone.** *Parasit Vectors* 2012, **5:**232.

13. Bridges DJ, Miller JM, Chalwe V, Moonga H, Hamainza B, Steketee R, Silumbe K, Nyangu J, Larsen DA: **Community-led Responses for Elimination (CoRE): a study protocol for a community randomized controlled trial assessing the effectiveness of community-level, reactive focal drug administration for reducing Plasmodium falciparum infection prevalence and incidence in Southern Province, Zambia.** *Trials* 2017, **18:**511.

14. Castellani J, Mihaylova B, Ajayi IO, Siribie M, Nsungwa-Sabiiti J, Afonne C, Serme L, Balyeku A, Kabarungi V, Kyaligonza J, et al: **Quantifying and Valuing Community Health Worker Time in Improving Access to Malaria Diagnosis and Treatment.** *Clin Infect Dis* 2016, **63:**S298-S305.

15. Medzihradsky OF, Kleinschmidt I, Mumbengegwi D, Roberts KW, McCreesh P, Dufour MK, Uusiku P, Katokele S, Bennett A, Smith J, et al: **Study protocol for a cluster randomised controlled factorial design trial to assess the effectiveness and feasibility of reactive focal mass drug administration and vector control to reduce malaria transmission in the low endemic setting of Namibia.** *BMJ Open* 2018, **8:**e019294.

16. von Seidlein L, Peto TJ, Landier J, Nguyen TN, Tripura R, Phommasone K, Pongvongsa T, Lwin KM, Keereecharoen L, Kajeechiwa L, et al: **The impact of targeted malaria elimination with mass drug administrations on falciparum malaria in Southeast Asia: A cluster randomised trial.** *PLoS Med* 2019, **16:**e1002745.

17. Cook J, Xu W, Msellem M, Vonk M, Bergstrom B, Gosling R, Al-Mafazy AW, McElroy P, Molteni F, Abass AK, et al: **Mass screening and treatment on the basis of results of a Plasmodium falciparum-specific rapid diagnostic test did not reduce malaria incidence in Zanzibar.** *J Infect Dis* 2015, **211:**1476-1483.

18. Scott CA, Yeshiwondim AK, Serda B, Guinovart C, Tesfay BH, Agmas A, Zeleke MT, Guesses GS, Ayenew AL, Workie WM, et al: **Mass testing and treatment for malaria in low transmission areas in Amhara Region, Ethiopia.** *Malar J* 2016, **15:**305.

19. Silumbe K, Yukich JO, Hamainza B, Bennett A, Earle D, Kamuliwo M, Steketee RW, Eisele TP, Miller JM: **Costs and cost-effectiveness of a large-scale mass testing and treatment intervention for malaria in Southern Province, Zambia.** *Malar J* 2015, **14:**211.

20. Newby G, Hwang J, Koita K, Chen I, Greenwood B, von Seidlein L, Shanks GD, Slutsker L, Kachur SP, Wegbreit J, et al: **Review of mass drug administration for malaria and its operational challenges.** *Am J Trop Med Hyg* 2015, **93:**125-134.

21. WHO: **Mass drug administration for falciparum malaria. A practical field manual.** 2017.

22. Altea Cico BJ: **PMI IRS Country Programs: 2017 Comparative Cost Analysis. PMI Africa Indoor Residual Spraying Project, Abt Associates Inc.** May 2018.

23. Johns B, Yihdego YY, Kolyada L, Dengela D, Chibsa S, Dissanayake G, George K, Taffese HS, Lucas B: **Indoor Residual Spraying Delivery Models to Prevent Malaria: Comparison of Community- and District-Based Approaches in Ethiopia.** *Glob Health Sci Pract* 2016, **4:**529-541.

24. WHO: **Indoor residual spraying: An operational manual for IRS for malaria transmission, control and elimination. Second edition.** 2015.

25. White MT, Conteh L, Cibulskis R, Ghani AC: **Costs and cost-effectiveness of malaria control interventions--a systematic review.** *Malar J* 2011, **10:**337.

26. Cico AJ, B.: **PMI IRS Country Programs: 2017 Comparative Cost Analysis.** In *PMI Africa Indoor Residual Spraying Project*. Rockville, MD: Abt Associates Inc.; 2018.

27. Zelman BW, Baral R, Zarlinda I, Coutrier FN, Sanders KC, Cotter C, Herdiana H, Greenhouse B, Shretta R, Gosling RD, Hsiang MS: **Costs and cost-effectiveness of malaria reactive case detection using loop-mediated isothermal amplification compared to microscopy in the low transmission setting of Aceh Province, Indonesia.** *Malar J* 2018, **17:**220.

28. Stansfield SK, Walsh J, Prata N, Evans T: **Information to Improve Decision Making for Health.** *Disease Control Priorities in Developing Countries, 2nd Edition* 2006**:**1017-1030.

29. Fitzpatrick C, Fleming FM, Madin-Warburton M, Schneider T, Meheus F, Asiedu K, Solomon AW, Montresor A, Biswas G: **Benchmarking the Cost per Person of Mass Treatment for Selected Neglected Tropical Diseases: An Approach Based on Literature Review and Meta-regression with Web-Based Software Application.** *PLoS Negl Trop Dis* 2016, **10:**e0005037.

30. Pitt C, Ndiaye M, Conteh L, Sy O, Hadj Ba E, Cisse B, Gomis JF, Gaye O, Ndiaye JL, Milligan PJ: **Large-scale delivery of seasonal malaria chemoprevention to children under 10 in Senegal: an economic analysis.** *Health Policy Plan* 2017, **32:**1256-1266.

31. Gilmartin CC, D.: **The costs of Seasonal Malaria Chemoprevention (SMC) in the Sahel Sub-Region of Africa: A Multi-Country Cost Analysis of the ACCESS-SMC project.** Management Sciences for Health; 2016.
